# A Systematic Review and Meta-Analyses of Interventional Clinical Trial Studies for Gene Therapies for the Inherited Retinal Degenerations (IRDs)

**DOI:** 10.3390/biom11050760

**Published:** 2021-05-19

**Authors:** Gearóid P. Tuohy, Roly Megaw

**Affiliations:** 1 MRC Human Genetics Unit, University of Edinburgh, Western General Hospital, Edinburgh EH4 2XU, UK; Roly.Megaw@ed.ac.uk; 2 Princess Alexandra Eye Pavilion, NHS Lothian, Edinburgh EH3 9HA, UK

**Keywords:** IRDs, retinitis pigmentosa, Leber congenital amaurosis (LCA), gene therapy, RCT, clinical trial, visual acuity (VA), mobility, multi-luminance mobility testing (MLMT), full-field stimulus testing (FST)

## Abstract

IRDs are one of the leading causes of visual loss in children and young adults. Mutations in over 271 genes lead to retinal dysfunction, degeneration and sight loss. Though no cure exists, gene augmentation therapy has brought hope to the field. This systematic review sought to assess the efficacy of available gene therapy treatments for IRDs. Databases and public resources were searched for randomised controlled trials (RCTs) and non-randomised studies of interventions (NRSIs). Standard methodological procedures were used, including a risk-of-bias assessment. One RCT and five NRSIs were assessed, all for adeno-associated virus two (AAV2)-mediated treatment of RPE-specific 65 kDa (RPE65)-associated LCA (Leber congenital amaurosis). Five outcomes were reported for meta-analyses. Modest improvements in visual acuity, ambulatory navigation/mobility testing or central retinal thickness was observed. There was significant improvement in red and blue light full-field stimulus testing (FST) (red light risk ratio of 1.89, treated v control, *p* = 0.04; and blue light risk ratio of 2.01, treated v control, *p* = 0.001). Study design assessment using a ROBIN-I tool (Cochrane Library) showed risk-of-bias judgement to be “*low/moderate*”, whilst there were “*some concerns*” for the RCT using a RoB-2 tool (Cochrane Library). Although comparison by meta-analysis is compromised by, amongst other issues, a variable amount of vector delivered in each trial, FST improvements demonstrate a proof-of-principle for treating IRDs with gene therapy.

## 1. Introduction

The IRDs are a heterogenous group of overwhelmingly monogenic eye conditions that cause premature sight loss [1]. Currently, 271 causal genes have been identified (last updated 8 April 2021 [2]) which have roles in various aspects of photoreceptor and/or retinal pigment epithelium (RPE) function. Dominant, recessive, X-linked, digenic and mitochondrial modes of inheritance have all been described [3,4,5,6,7,8,9,10,11,12,13,14]. IRDs cause progressive retinal degeneration, which results in a variety of progressive symptoms including night blindness (nyctalopia), visual field constriction, central visual loss, dark adaptation problems, photophobia, nystagmus and pupillary abnormalities [14].

There is considerable phenotypic variability between IRDs and historically they have been grouped into several different disease patterns, including retinitis pigmentosa (RP), cone dystrophies, cone-rod dystrophies and Leber congenital amaurosis (LCA) [15]. Further, many attempts have been made at genotype-phenotype correlation. However, in reality, there is both considerable inter-allelic disease overlap and marked intra-allelic disease variability. Thus, the progress made in genetic diagnosis of IRDs has been invaluable. 

LCA is a severe congenital or early infant-onset IRD characterised by vision loss, nystagmus, an absence of a normal pupil response and an almost non-recordable ERG [16,17,18,19]. Known genes associated with LCA includes *GUCY2D* (estimated 10–20% patients), *CEP290* (15–20%), *CRB* (10%), *AIPL1* (4–8%) and *NMNAT1* (uncertain). Following the original description of the infantile disorder, a subsequent milder form of disease, considered to be on the LCA spectrum, was described that presents in the 6th or 7th year of life and leads to blindness by the age of 30 [20]. Whilst this later-onset disease has been referred to by several different names [21,22,23,24,25], there is considerable overlap with LCA in both genotype and phenotype, with causal genes including *RPE65* (5–10%), *LRAT* (<1%) and *RDH12* (4–5%) [18].

Mutations in *RPE65* are estimated to account for approximately 5–10% of LCA and approximately 1–2% of retinitis pigmentosa [26]. *RPE65* is localized to chromosome 1p31, comprising 14 exons and encoding a 65-Kd protein [23]. *RPE65* is a key component of the retinoid visual cycle. It is expressed in the retinal pigment epithelium (RPE) which, together with LRAT, is involved in continuous regeneration of the visual chromophore [24]. LCA-mediated IRDs have a prevalence of between 1 in 33,000 [25] and 1 in 81,000 [27]. In Ireland, there are an estimated ~130 LCA patients [28], while NICE has reported there may be 86 LCA2 patients potentially eligible for EMA-approved gene augmentation therapy [29] in England [30].

Gene augmentation therapy is a novel therapeutic approach for genetic disease that seeks to replace null or loss-of-function protein by expressing wild-type copies of the gene of interest, typically through delivery via a viral capsid [31]. The approach is most applicable to recessive traits, and decades-long efforts have demonstrated the approach efficient at rescuing visual loss in animal models of achromatopsia, X-linked and recessive RP, LCA and Stargardt’ disease, amongst others [32,33].

Subsequently, gene augmentation therapy has begun to be translated in clinical trials. ClinicalTrials.gov have estimated 250 listed studies focused on IRDs, including patient registry studies, natural histories, observational and interventional trials [34]. One of the first genes to be targeted was *RPE65*-LCA, so chosen because of the relative delay in the development of retinal degeneration despite early-onset visual loss, thus offering a wide treatment window. Gene augmentation therapy (voretigene neparvovec-rzyl/*Luxturna* [35]) has now received FDA approval in the USA (2017) and EMA approval in Europe (2018) for adult and paediatric disease. This first-in-class treatment gives the field hope that a new class of drugs may arise for IRDs [36,37].

Gene augmentation therapies used to date to treat IRDs are based around similar basic adeno-associated virus (AAV) vectors and their capsids, with a variety of promoters chosen by different research groups and companies. To rigorously determine the efficacies of these new therapies it is critical to assess how results show benefit. This requires that methodology, study design and outcome measures provide a clear and reasonable conclusion for the impact on the patient. To this end, we performed a systematic review and meta-analyses of clinical trials for RP patients undergoing gene therapy.

## 2. Materials and Methods 

### 2.1. Criteria for Considering Studies for This Review

#### 2.1.1. Types of Studies

Articles eligible for inclusion in this systematic review were interventional clinical trials, either randomized or non-randomized, for gene therapy treatments for IRD patients, published in English searched in the relevant databases from <1946 to 2020 Week 5>.

#### 2.1.2. Types of Participants

All patients who have been diagnosed with IRDs, either non-syndromic or syndromic, were included with no restrictions of age, gender or ethnicity. Clinical trials were excluded from patients with ocular comorbidities, or excluded from patients with complications known to influence visual function. Women who were pregnant or lactating or any participants unwilling to use effective contraception were also ineligible.

#### 2.1.3. Types of Interventions

Studies included any investigational gene therapy interventions for IRDs. There were no comparators available for any approved interventions for IRD patients.

### 2.2. Types of Outcome Measures

#### 2.2.1. Primary Outcomes

The primary outcome of intervention was a mean change from baseline best corrected visual acuity (BCVA) at one year, as measured by the Early Treatment Diabetic Retinopathy Study (ETDRS) chart and measured by logMAR (standard logarithm of the minimum angle of resolution) [38,39,40,41].

Ambulatory navigation/mobility mazes have been developed by a number of research groups and were included as a primary outcome. However, several methodologies exist. To allow comparison between trials, reporting of these assays used a mean difference, i.e., comparing the proportion of improved performances post-operatively, between groups (a risk ratio [RR]).

#### 2.2.2. Secondary Outcomes

Secondary outcomes included full-field light sensitivity threshold (FST) [42], visual field, visual perception, electroretinogram (ERG), Goldmann visual fields, fundus photography, nystagmus testing, central retinal thickness (as measured by optical coherence tomography (OCT)), pupillary light reflex response (PLR) and diagnostic ophthalmic techniques [38,43,44].

#### 2.2.3. Adverse Events

Adverse events were not searched for vector administration, due to a considerable volume of literature for AAV safety outcomes from several authors [45,46,47,48,49].

### 2.3. Search Methods for Identification of Studies

#### 2.3.1. Electronic Searches

The literature search used the Cochrane Handbook for Systematic Reviews of Interventions [50], using the Ovid database for MEDLINE and EMBASE.

We used a PICOS strategy to identify a systematic review of interventional clinical trials (*the study design*) for gene therapies (*the intervention*) for IRD patients (*the population*), for the purpose of improving the disorder (*the outcomes*), given there were no treatments available (*the comparison*). The PICOS search terms and search strings included 36 words and phrases using MeSH terms and Boolean operators, identified in Table S1 (Supplemental).

Structuring and collection of the relevant studies used the PRISMA checklist process [51]. We used the ROBINS-I risk of bias in non-randomised studies of interventions tool [52,53] and the RoB-2 tool for the Cochrane Collaboration’s process for assessing risk of bias in randomised trials [54].

#### 2.3.2. Searching Other Resources

We searched FDA and clinicaltrials.gov databases, including the Biologics License Application resource (BLA) at FDA.

### 2.4. Data Collection and Analysis

#### 2.4.1. Selection of Studies

Following database searching, each article was assessed as being definitely relevant, possibly relevant or definitively not relevant. Duplicates were removed and all articles assessed for exclusion and inclusion. All included articles were evaluated for study design and reports and final studies reviewed in depth. 

#### 2.4.2. Data Extraction and Management

All relevant data (intervention characteristics, study design, primary and secondary outcomes) were extracted and collected in Excel prior to analysis [55] of all available data with Review Manager (RevMan) 5.4 software [56].

#### 2.4.3. Assessment of Risk of Bias in Included Studies

Selected studies were independently assessed for sources of systematic bias according to the guidelines in the relevant sections for the Cochrane Handbook for the Systematic Reviews of Interventions [50] using ROBINS-I and Rob-2 tools.

#### 2.4.4. Measures of Treatment Effect

Primary and secondary outcome data was assessed in accordance with the methods within each selected study. Number of BCVA letters/logMAR at one year (or more) was used to collect the mean difference (MD), standard deviations [SD] and corresponding 95% confidence intervals (CI), comparing mean change from baseline between groups. Continuous data was additionally recorded for improvements (with 95% CIs) for full-field stimulus testing data (red and blue wavelengths) and retinal thickness. Risk ratios (RRs) (with 95% CIs) for dichotomous outcomes were reported, including the proportion of participants with improved/worsening mobility/ambulatory navigation. A random-effects model in RevMan 5.4 meta-analysis [57] was used for meta-analysis. 

#### 2.4.5. Unit of Analysis Issues

For most studies, the unit of analysis was the individual participant (one study eye per participant). Five of six studies used a design with one treated eye compared to an untreated control eye. One study (Russell, 2017 [58]), used a crossover design where both eyes received interventions one year apart.

#### 2.4.6. Missing Data

Missing data was not imputed for the purpose of the analysis while only one study (Russell, 2017 [58]) used both an intent-to-treat (ITT) and a modified intent-to-treat (mITT) model.

#### 2.4.7. Heterogeneity

Heterogeneity was tested between the studies using chi-square analysis with significant heterogeneity (*p* < 0.05) precluding meta-analysis. An I^2^ value of greater than 50% indicated a substantial statistical heterogeneity.

#### 2.4.8. Assessment of Reporting Biases

ROBIN-I and ROB tools were used to assess risk of bias in the five NSRIs and one RCT respectively. Assessments were made by 2 independent examiners. 

## 3. Results 

### 3.1. Systematic-Review of Search Results

**Figure 1:**
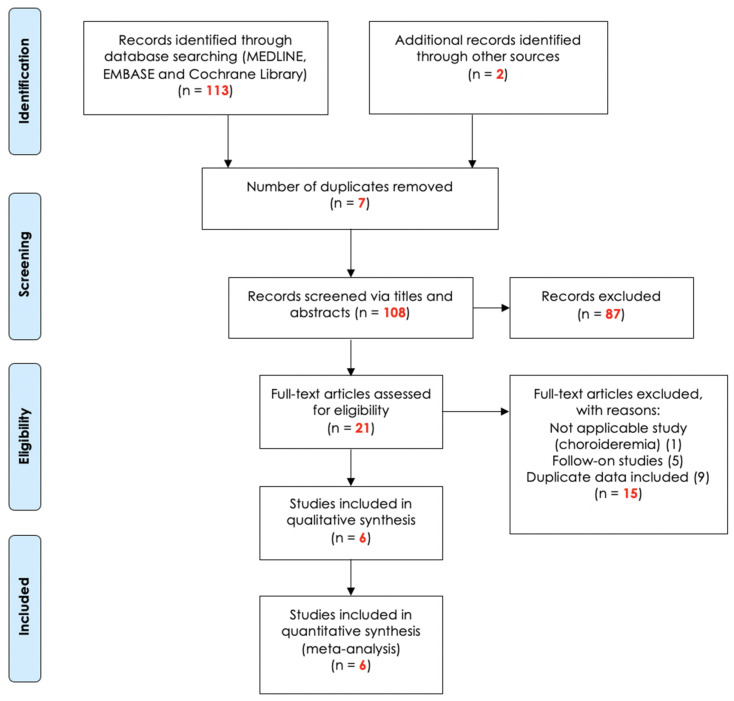
PRISMA flowchart of 115 studies identified, excluded, reviewed and selected.

Following the structured search approach (Appendix A.1), 115 peer-reviewed research articles were screened and assessed (Figure 1; Appendix A.2), seven articles were removed due to duplication, leaving one hundred and eight articles to be screened. Eighty-seven records were excluded that did not include relevant information. Twenty-one articles were accessed for eligibility; fifteen articles were excluded: one was not applicable for meta-analysis (a study on gene therapy for choroideremia), five were follow-up studies and nine articles included duplicate data. This left six final articles (Appendix A.3) for review and meta-analysis:

### 3.2. Outcomes

**Figure 2:**
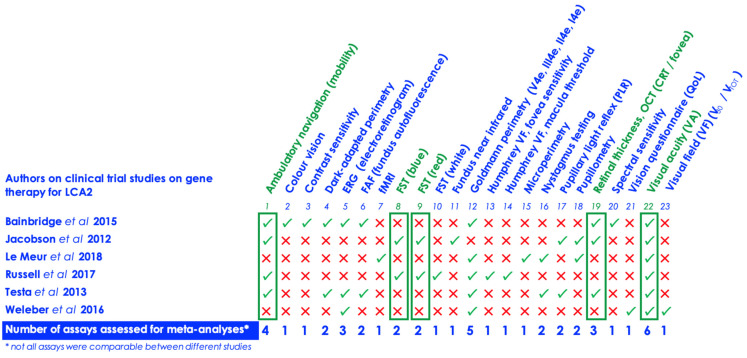
A summary of the assays collated from the six (6) studies, arranged alphabetically.

In total, 23 different assays were reported and analysed across the six studies in Figure 2 and Table 1 (including Figure S1a–c, Appendix A.4 and Table A1). Safety data was not collected on specific AAV2 vectors, having been examined in other independent studies on interventional clinical studies in the retina [37,59,60,61,62]. Only one assay, visual acuity (VA), was common to all six papers. Of the 23 assays reviewed, only five outcomes were reported for meta-analysis—VA (logMAR), mobility, red light full-field stimulus (FST) testing (log10(cd.s/m^2^)), blue light full-field stimulus (FST) testing (log10(cd.s/m^2^)) and central retinal thickness (CRT).

All continuous and dichotomous data was reported. If either continuous and dichotomous data were available, then analysis was used to compare and contrast the models. However, continuous data was preferred from a statistical perspective as some information risked being lost in categorical data.

### 3.3. Visual Acuity Measured by logMAR 

**Figure 3:**
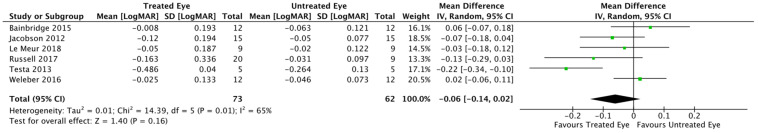
Meta-analysis shows no significant improvement in visual acuity following treatment. RevMan analysis of logMAR visual acuity, using a random effects model and summary statistic for continuous data, shows a modest improvement (mean difference −0.06 [−0.14, 0.02]) that does not reach statistical significance (*p* = 0.16).

A 0.30 logMAR (3 line) mean post-operative change of VA was accepted as being a “clinically meaningful” improvement [43]. VA results were reported in Figure 3. Overall, outcomes showed a benefit of treatment compared to control eyes, but did not meet statistical significance. 

There was a mean [SD] improvement of −0.142[0.181] logMAR letters in treated eyes (*n* = 73), compared to −0.079[0.103] logMAR letters in untreated (*n* = 62), showing a difference of logMAR −0.063 (including Table S2 (Supplemental)). RevMan 5.4 analysis showed a statistical difference of −0.06 logMAR (95% CI [−0.14, 0.02], *p* = 0.16) above. Individually, only one study (Testa, 2013), reported a clinically meaningful improvement in VA, with a mean [SD] improvement of −0.49 [0.04] logMAR letters in the treated eye, compared to a mean [SD] improvement of −0.26 [0.13] logMAR in the untreated eye.

Finally, an analysis of dichotomous data on visual outcomes post treatment (better or worse) was performed. Five studies provided individual patient data to allow this analysis (*n* = 58 treated eyes; *n* = 47 untreated eyes). The line of no effect showed an RR of 1.13 (95% CI 0.83, 1.53), indicating an improvement with treatment that did not reached clinical significance (*p* = 0.44) (Figure S2 (Supplemental)).

### 3.4. Mobility 

**Figure 4:**
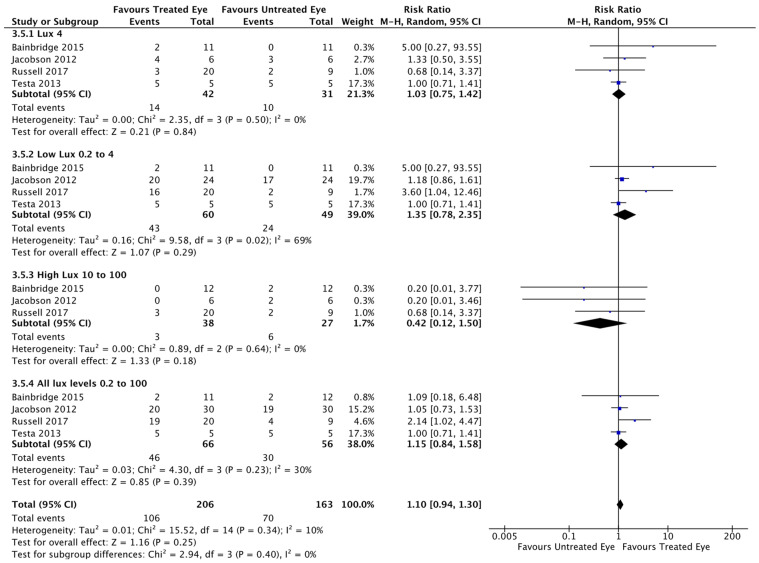
Meta-analysis shows no significant improvement in ambulatory navigation/mobility following treatment. RevMan meta-analysis of dichotomous data showed no significant improvement in performance across all light intensities analysed.

Given the disparity between the four different mobility methods used in the studies in terms of size, light intensity, scoring and reporting, no direct comparison was possible. Instead, a meta-analysis of dichotomous data (better/worse post-treatment) was performed, To do so, four sub-groups were defined, according to light intensity used to illuminate the mobility mazes: (a): mobility under a single light of intensity of 4 lux; (b): mobility under a “low” ambient light level (0.2, 0.6, 1, 2 or 4 lux), broadly scotopic light; (c): mobility under a “high” ambient light level (10, 15, 50, 100 or 125 lux), broadly photopic light vision function; (d): mobility under all ambient light levels ranging from 0.2 to 100 lux. Results are summarised in Figure 4 (and in Table S3 (Supplemental)).

Under a light intensity of 4 lux (Figure 4, “*Lux 4*”), analysis of 4 studies showed an RR of 1.03 (95% CI 0.75, 1.42), indicating an improvement with treatment that did not reach clinical significance (*p* = 0.84). Under low ambient light (“*Low Lux 0.2 to 4*”), analysis of 4 studies showed an RR of 1.35 (95% CI 0.78, 2.35), indicating an improvement with treatment that did not reach clinical significance (*p* = 0.29). Under high ambient light (“*High Lux 10 to 100*”), analysis of 4 studies showed an RR of 0.42 (95% CI 0.12, 1.50), indicating a worsening with treatment that did not reach clinical significance (*p* = 0.18). Analysis of all ambient light levels (“*All lux levels 0.2 to 100*”) of 4 studies showed an RR of 1.15 (95% CI 0.84, 1.58), indicating an improvement with treatment that did not reach clinical significance (*p* = 0.39).

### 3.5. Full-Field Stimulus (FST) Testing for Red and Blue Wavelength

**Figure 5:**
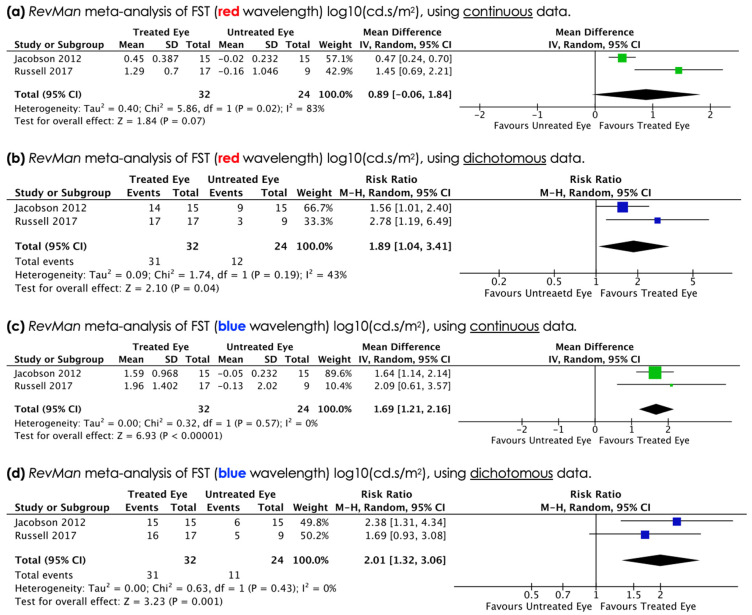
Meta-analysis shows significant improvement in full field sensitivity in response to red and blue light (log10(cd.s/m^2^) following treatment [(**a**–**d**)]. RevMan analysis of dichotomous FST data shows significant improvement with red (RR1.89; *p* = 0.04) and blue (RR 2.01; *p* = 0.001) stimuli. Continuous data shows improvement with blue (mean difference 1.69, *p* < 0.00001) but not red (mean difference 0.89, *p* = 0.07) light.

Only two studies used FST testing that allowed for a meta-analysis. Both continuous and dichotomous (better/worse) data was analysed (Figure 5a–d).

Under red light FST results, analysis of continuous data, showed a mean difference [MD] of 0.89 log10(cd.s/m^2^) (95% CI −0.06, 1.84) in treated eyes compared to control, indicating an improvement with treatment that did not reach clinical significance (*p* = 0.07). Analysis of dichotomous data (better/worse) for red light FST showed a RR of 1.89 (95% CI 1.04, 3.41), indicating an improvement with treatment that reached clinical significance (*p* = 0.04). 

Under blue light FST results, analysis of continuous data, showed a difference of 1.69 log10(cd.s/m^2^) (95% CI 1.21, 2.16) in treated eyes compared to control, indicating an improvement with treatment that reached clinical significance (*p* = 0.00001). Analysis of dichotomous data (better/worse) showed a RR of 2.01 (95% CI 1.32, 3.06), indicating an improvement with treatment that reached clinical significance (*p* = 0.001).

### 3.6. Central Retinal Thickness (CRT) 

**Figure 6:**
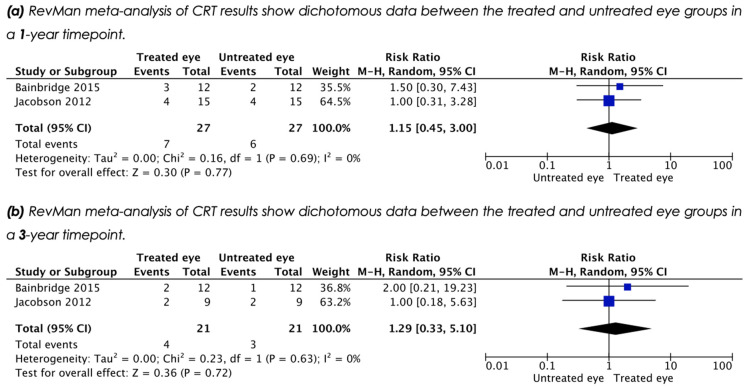
Meta-analysis shows no significant improvement in central retinal thickness following treatment [(**a**,**b**)]. RevMan meta-analysis of dichotomous data showed no significant improvement in CRT measurement.

Three studies reported CRT outcomes as measured by optical coherence tomography (OCT), but only two included quantitative data that allowed for meta-analysis. Analysis of dichotomous data (thinner/thicker) at 1 year post treatment, showed a RR of 1.15 (95% CI 0.45, 3.00), indicating an increase of CRT with treatment that did not reach clinical significance (*p* = 0.77) (Figure 6a). Analysis of dichotomous data for a long term timepoint (3 years), showed a RR of 1.29 (95% CI 0.33, 5.10), indicating an increase of CRT with treatment that did not reach clinical significance (*p* = 0.72) (Figure 6b).

### 3.7. Risk of Bias Tools within Studies

Cochrane risk-of-bias tools were used to assess study reliability; ROBIN-I methods [52], for non-randomised study designs, and RoB-2 methods [63], for randomised clinical trials. Overall, a risk-of-bias judgement was reported “low/moderate”, with a predicted direction of bias “towards null/unpredictable” for the 5 NRSIs, and a report with “some concerns” and a predicted direction of bias with “favours experimental” for the RCT (Table 2; Appendix A.5, Table A2. and Appendix A.6, Table A3).

Finally, Table 3 provided a summary of 12 meta-analyses reported for each of the outcomes, a PRISMA summary of a structured abstract in Appendix A.7, and a PRISMA checklist in Appendix A.8 (Table A4). 

## 4. Discussion

Inherited retinal dystrophies (IRDs) are a leading cause of visual loss in children and adults of working age. Formerly untreatable, the emergence of gene augmentation therapy represents a real paradigm shift in patient care. We thus performed a systematic review and meta-analysis of interventional clinical trials to assess the efficacy of gene therapies for IRDs, thus delivering useful information for both clinicians and patients. The purpose of this systematic review is also based on a “fair test” [64], grounded in evidence-based medicine [65,66] (and Figure S1). To test such new therapies, it is critical to assess how transparent results show clear benefit for the patient. This requires that methodology, study design and outcome measures have to provide a clear and reasonable conclusion for the impact on the patient. A systematic review and meta-analysis of IRD patient outcomes for gene therapy is critical in order to support the field [67]. 

A search of peer-reviewed literature found that only gene therapies to treat Leber congenital amaurosis (LCA) met the criteria for addressing the original question (Appendix A.1). LCA is a rare disorder and gene therapy is an expensive treatment, which led to studies with small patient numbers. Further, the particularly severe phenotype of the disease, with low visual acuity from birth, led to difficulties in assessing the effect of treatment. 

Of the 6 studies analysed, a significant drawback to the meta-analysis performed here is the variability in vector design and concentration of virus injected sub-retinally. All studies analysed used an AAV2 serotype, with most using an AAV2/2 capsid. However, one study used an AAV2/4 capsid. Further, some studies used a hybrid chicken β-actin promoter with a cytomegalovirus enhancer, whilst some used the human RPE65 promoter. Treatment doses ranged from 10^8^ to 10^12^ vg, in volumes from 0.15 to 1.0 mL. This spans a number of logarithmic steps in each dose, potentially compromising the comparison of the results within the 6 studies. Despite all this, in our view, the similarities in products compared in the meta-analyses outweigh the differences. All contain the same recombinant human *RPE65* gene, all are packaged in a similar AAV2 vector and all use a similar sub-retinal surgical procedure for delivery. All were used to treat the same trial population (RPE65-LCA2 patients). Further, there were similar criteria for controls and there was considerable overlap in trial duration and endpoints. Finally, we felt the comparison appropriate as pre-clinical work has shown good photoreceptor transduction and expression efficiency. As such, despite the analyses’ obvious limitations, we felt it appropriate in order to increase numbers of this rare disease and thus improve statistical power. Given the differences outlined, it is extremely encouraging to note the significant improvements in full-field stimulus (FST) testing that are seen following meta-analysis.

With gene augmentation in its infancy, it is perhaps unsurprising that there were variabilities in the biomarkers used to determine treatment efficacy. In total, 23 outcome assays were used. Visual acuity was the only outcome used in all six studies analysed. Five studies used Goldmann perimetry, four used ambulatory navigation/mobility and three used electroretinography. A further nineteen assays were used in two or less studies. Many of the assays were not comparable for several reasons. Five studies assessed visual fields using Goldmann perimetry, although different studies presented different isopters with variable follow up time. Further, a lack of quantitative data in some studies meant overall meta-analysis was not possible. Three studies used electroretinography as an assay, but two provided no data. Other assays used in two or more studies were unsuitable for meta-analysis due to a lack of quantitative data or irreconcilable differences in the way data was presented. As the field evolves, it is hoped agreed standards for methods and reporting will be established, allowing for easier meta-analyses of trials.

Visual acuity is the gold standard assay by which retinal disease treatments are assessed. Though our meta-analysis showed only modest improvement with gene therapy (in terms of clinical or statistical significance), the result is perhaps not surprising given the low-vision phenotype of LCA patients. Two studies (Bainbridge and Testa) did not provide raw visual acuity data and instead patient vision was determined from results presented in study graphs. This was undertaken by two independent researchers, with a mean of the two readings being used, but an element of uncertainty remains with the overall result due the unavailability of raw data within the actual papers. It should be noted that an I^2^ value 65% indicates substantial statistical heterogeneity within the VA assays (Figure 3). As such, little weight can be placed upon the outcome of our VA analyses.

The study designs often dictated that the eye with the worse vision was treated, with control eyes having a better baseline vision. Although logMAR vision charts determine a linear improvement in vision with each letter or line gained, if treatment and control arms have different baseline values, bias is introduced and outcomes may be influenced as a result. Without adjustment, it may be unclear what impact arises from the treatment effect, as opposed to the treated eye being worse at baseline. Even adjusted data may not be robust enough to eliminate this confounding factor. Emerging gene therapy trials, where both eyes are treated and compared for one year to a deferred treatment group, should address this issue. 

Four studies reported mobility testing as a key outcome. Mobility testing for the MLMT assay (Russell et al.) received criticism by an independent commentator [68] and reviewers in the FDA regarding uneven luminance levels [29]. In addition, we note that the MLMT assay results were indirect. A “passing level” of the assay compared baselines between 1-year timepoints however, the original data for measuring speed, time, accuracy (and further components) for assessment, were not included in the paper or the Biologicals License Application (BLA). Further, the MLMT assay used a logarithmic scale, based on light intensity (lux), which was then subsequently converted to an ordinal scale (ranging from −1 to 6), such that a two-point change in the ordinal scale may have a different interpretation depending on the baseline score (Table S4 (Supplemental)) [68]. 

Due to disparate methodologies (maze size and design, measurement, quantification and reporting), only analysis of dichotomous data (better/worse post treatment) was possible. This risks overestimating the benefit in certain studies. For example, Russell et al. reasoned that results in their maze required at least 2 levels of improvement on their assessment scale to accept the result as showing therapeutic benefit, whereas we defined even a 1 level gain post-operatively as performing ‘better’. 

Some studies had datapoints missing, while quantitative data was missing from others, and required interpretation from results presented in study graphs (Jacobson et al.). Though RevMan analysis of dichotomous data suggested overall improvement in mobility, statistical significance was not reached. At present, there is no better test available for assessing the impact of gene therapy on visual function and so, as the field develops, it would be advantageous if some standardisation of the test could be agreed upon, recently supported by other literature [69,70,71]. 

The use of full-field stimulus (FST) testing (white, red and blue wavelength) is highly relevant because few research tools can quantify changes in visual perception if sight loss is as severe as it is in an RPE65/LCA2 population. Thus, the FST data carries extra significance. FST results presented in the studies was at times confusing. One study (Russell et al.) alternately presented white light FST results in log10(cd.s/m^2^) units and −log10(cd.s/m^2^) units, whilst not commenting on their red and blue light FST results (Russell et al.). A further study only described results in terms of “log10” units, which we interpreted as log10(cd.s/m^2^) units (Jacobson et al.), thus allowing for meta-analysis. Although the FDA, as part of the Biologics Licence Application (BLA) review for Luxturna [29], stated ‘*the direct clinical benefit of FST is not clear’,* it is apparent from meta-analysis of these two studies that retinal sensitivity improves with AAV-mediated gene augmentation therapy for RPE65-mediated LCA2. The significance of this cannot be underestimated. It is proof of principle that visual improvement is achievable with this technology and gives us hope that similar benefit could be achieved when other alleles are targeted. 

The first attempt to use gene augmentation therapy for retinal disease has led to an FDA and EMA approved product (voretigene neparvovec-rzyl [*Luxturna*]). Improvements in surgical technique and improved knowledge of treatment technicalities (e.g., virus concentration) could mean subsequent iterations of these therapies show improved efficacy. Further, the more novel outcomes for mobility may drive innovative end-points. Though some concerns were raised by our Cochrane risk of bias analysis, further treatments targeting more common disease-causing genes [37] will mean increased patient numbers in trials and may allow for blinded evaluations, resulting in more robust studies.

As of April 2021, there are > 40 interventional gene therapy trials for IRDs reported at clinicaltrials.gov, from both academic and commercial institutions targeting several different IRD genes [72,73,74,75,76]. This meta-analysis highlights the need for consistency of trial design to allow comparison of gene products, but also shows the potential this technology has for addressing a leading cause of blindness in children and adults of working age.

## 5. Conclusions

The objective of this work was to conduct a systematic review of interventional clinical trial studies for IRDs and to assess and compare the effectiveness of available gene therapy treatments. Following the search, review and analysis of the relevant studies, the systematic review concluded that a meta-analysis for AAV-RPE65 gene therapy for LCA2 reported a modest improvement for visual acuity, mobility and full-field stimulus testing (FST), with FST improvements reaching statistical significance. In terms of a recommendation to support the IRD patient communities and researchers, we propose that full and open-access data is key. If the field is to be progressed and improved, then objective and transparent results need to be shared in order to improve outcomes, analysis, reporting and interpretation.

## Figures and Tables

**Table 1:** Study selection of six (6) research articles for review and meta-analysis outcomes for LCA2-RPE65 gene therapy.

Studies (*n* = 6) (*Journal*)	Trial Identifier (ClinicalTrials.gov)	Study Type/Viral Vector	Titre (vg ^(a)^)/Injection Vol.	Population (ITT ^(b)^)	Age Range (in Years)	BCVA ^(c)^ (logMAR)	Ambulatory Navigation, Low Light Ambient Level (<4 lux) (RR ^(d)(e)^)	FST ^(f)^ (Red Light), log10(cd.s/m^2^)	FST ^(f)^ (Blue Light), log10(cd.s/m^2^)	Retinal Thickness (OCT, µm)
**Bainbridge et al., 2015** *(NEJM)*	NCT00643747	Phase 1–2, open-label, non-randomized; rAAV 2/2. hRPE65p.hRPE65	1.0 × 10^11^ to 1.0 × 10^12^ vg; injection volume of 900 µL to 1 mL	12	**6–23 years** *(Median, 15y; Mean, 14.4y; CI 95%, 3.8)*	Mean change BCVA of **−0.008** logMAR (treated eyes) vs. **−0.063** logMAR (untreated eyes), a difference of **0.06** logMAR (95% CI −0.14, 0.02)	Risk ratio of **5.00** (95% CI 0.27, 93.55)	*Data unavailable*	*Data unavailable*	Risk of ratio of **1.50** (95% CI 0.30, 7.43)
**Jacobson et al., 2012** *(Arch Ophthalmol)*	NCT00481546	Phase 1, open-label, non-randomized; rAAV2-RPE65	5.96 × 10^10^ to 17.88 × 10^10^ vg; injection volume of 150 µL to 450 µL	15	**11–30 years** *(Median, 20y; Mean, 19.6y; CI 95%, 3.1)*	Mean change BCVA of **−0.12** logMAR (treated eyes) vs. **−0.05** logMAR (untreated eyes), a difference of **−0.07** logMAR (95% CI −0.18, 0.04)	Risk ratio of **1.18** (95% CI 0.86, 1.61)	Mean change FST of **0.45** log10(cd.s/m^2^) (treated eyes) vs. **−0.02** log10(cd.s/m^2^) (untreated eyes), a difference of **0.47** log10(cd.s/m^2^) (95% CI 0.24, 0.70)	Mean change FST of **1.59** log10(cd.s/m^2^) (treated eyes) vs. **−0.05** log10(cd.s/m^2^) (untreated eyes), a difference of **1.64** log10(cd.s/m^2^) (95% CI 1.14, 2.14)	Risk of ratio of 1.00 (95% CI 0.31, 3.28)
**Le Meur et al., 2018** *(Mol Ther)*	NCT01496040	Phase 1/2, open, non-randomized; AAV2/4.-RPE65-RPE65	1.22 × 10^10^ to 4.8 × 10^10^ vg; injection volume of 200 µL to 800 µL	9	**9–42 years** *(Median, 22y; Mean, 24.1y; CI 95%, 7.8)*	Mean change BCVA of **−0.05** logMAR (treated eyes) vs. **−0.02** logMAR (untreated eyes), a difference of **−0.03** logMAR (95% CI −0.18, 0.12)	*Data unavailable*	*Data unavailable*	*Data unavailable*	*Data unavailable*
**Russell et al., 2017** *(Lancet)*	NCT00999609	Phase 3, open-labelled, randomised (RCT); AAV2-hRPE65v2	1.5 × 10^11^ vg; injection volume of 300 µL	31	**4–44 years** *(Median, 11y; Mean, 14.4y; CI 95%, 4.1)*	Mean change BCVA of **−0.163** logMAR (treated eyes) vs. **−0.031** logMAR (untreated eyes), a difference of **−0.13** logMAR (95% CI −0.29, 0.03)	Risk ratio of **3.6** (95% CI 1.04, 12.46)	Mean change FST of **1.29** log10(cd.s/m^2^) (treated eyes) vs. **−0.16** log10(cd.s/m^2^) (untreated eyes), a difference of **1.45** log10(cd.s/m^2^) (95% CI 0.69, 2.21)	Mean change FST of **1.96** log10(cd.s/m^2^) (treated eyes) vs. **−0.13** log10(cd.s/m^2^) (untreated eyes), a difference of **2.09** log10(cd.s/m^2^) (95% CI 0.61, 3.57)	*Data unavailable*
**Testa et al., 2013** *(Ophthalmology)*	NCT00516477	Phase 1, open-label, non-randomized (3-year study); AAV2-hRPE65v2	1.0 × 10^8^ to 5.0 × 10^8^ vg; injection volume of 150 µL to 300 µL	5	**11–26 years** *(Median, 19y; Mean, 19.8y; CI 95%, 7.9)*	Mean change BCVA of **−0.486** logMAR (treated eyes) vs. **−0.264** logMAR (untreated eyes), a difference of **−0.22** logMAR (95% CI −0.34, −0.10)	Risk ratio of **1.0** (95% CI 0.71, 1.41)	*Data unavailable*	*Data unavailable*	*Data unavailable*
**Weleber et al., 2016** *(Ophthalmology)*	NCT00749957	Phase 1–2, open-label, non-randomized; rAAV2-CB-hRPE65	1.0 × 108 to 5.0 × 108 vg	12	**6–39 years** *(Median, 31y; Mean, 24.8y; CI 95%, 8.5)*	Mean change BCVA of **−0.025** logMAR (treated eyes) vs. **−0.046** logMAR (untreated eyes), a difference of **0.02** logMAR (95% CI −0.06, 0.11)	*Data unavailable*	*Data unavailable*	*Data unavailable*	*Data unavailable*
**Summary meta-analyses**		Phase 1, 1/2, 3; AAV2	**Range** from 1.0 × 10^8^ to 1.0 × 10^12^ vg; injection vol. 150 µL to 1 mL	**Population** n = 84	**Range** 4–44 years	**Summary** weighted mean difference (MD) of **−0.06** logMAR improvement over treated vs. untreated eye (95% CI −0.14, 0.02), *p* = 0.16	**RR** improvement of **1.35**, over treated vs. untreated eye (95% CI 0.78, 2.2.35), *p* = 0.29	**Summary** weighted mean difference (MD) of FST (red) **0.89** log10(cd.s/m^2^) over treated vs. untreated eye (95% CI −0.06, 1.84), *p* = 0.07	**Summary** weighted mean difference (MD) of FST (blue) **1.69** log10(cd.s/m^2^) over treated vs. untreated eye (95% CI 1.21, 2.16), *p* = 0.00001	**RR** improvement of **1.15** (95% CI 0.45, 3.00), *p* = 0.77

(a) vg—vector genomes; (b) ITT—intention to treat; (c) BCVA—Best corrected visual acuity, (logMAR); (d) RR—risk ratio; (e) 95% CI—95% confidence interval; (f) FST—full-field stimulus testing (red and blue wavelength), log10(cd.s/m^2^).

**Table 2:** Analysis of risk of bias studies for five (5) NRSIs and one (1) RCT.

Study Author & Year	ROBIN-I (Risk of Bias in Non-Randomised Studies of Interventions)		RoB-2 (Risk of Bias in Randomised Studies of Interventions [RCT])	
	Risk-of-Bias Judgement	Overall Predicted Direction of Bias	Risk-of-Bias Judgement	Overall Predicted Direction of Bias
Bainbridge et al., 2015	Low/Moderate	Towards null/Unpredictable	*N/A*	*N/A*
Jacobson et al., 2012	Low/Moderate	Towards null/Unpredictable	*N/A*	*N/A*
Le Meur et al., 2018	Low/Moderate	Towards null/Unpredictable	*N/A*	*N/A*
Russell et al., 2017	*N/A*	*N/A*	Some concerns	Favours experimental
Testa et al., 2013	Low/Moderate	Towards null/Unpredictable	*N/A*	*N/A*
Weleber et al., 2016	Low/Moderate	Towards null/Unpredictable	*N/A*	*N/A*

**Table 3:** Summary table of 12 meta-analyses with a total of all treated and untreated eyes showed that three meta-analyses have reported statistical significance within the table below *. Of the 3 of the 12 meta-analyses that reached statistical significance, FST (red light) had an RR improvement of 1.89 (95% CI 1.04, 3.41) *p* = 0.04; FST (blue light) had a MD improvement of 1.69 (95% CI 1.21, 2.16) *p* = 0.00001, and finally; FST (blue light) had an RR improvement of 2.01, (95% CI 1.32, 3.06), *p* = 0.001.

No.	Meta Analyses	Number of Studies for Meta-Analysis	Treated Eyes	Untreated Eyes	Study Author	Continuous (C)/Dichotomous (D)	Formal Result (MD or RR)	95% Confidence Interval	Chi^2^	I^2^	Z Efffect	*p* Value
**1**	LogMAR visual acuity	6	73	62	Bainbridge, Jacobson, Le Meur, Russell, Testa, Weleber	C	MD −0.06	CI (−0.14, 0.02)	14.39	65%	1.40	0.16
**2**	LogMAR visual acuity	6	58	47	Bainbridge, Jacobson, Le Meur, Russell, Testa, Weleber	D	RR 1.13	CI (0.83, 1.53)	3.92	0%	0.77	0.44
**3**	Ambulatory navigation/mobility: Sub-group A (4 lux)	4	42	31	Bainbridge, Jacobson, Russell, Testa,	D	RR 1.03	CI (0.75, 1.42)	2.35	0%	0.21	0.84
**4**	Ambulatory navigation/mobility: Sub-group B (0.2, 0.6, 1, 2, 4 lux)	4	60	49	Bainbridge, Jacobson, Russell, Testa,	D	RR 1.35	CI (0.78, 2.35)	9.58	69%	1.07	0.29
**5**	Ambulatory navigation/mobility: Sub-group C (10, 15, 50, 100, 125 lux)	3	38	27	Bainbridge, Jacobson, Russell	D	RR 0.42	CI (0.12, 1.50)	0.89	0%	1.33	0.18
**6**	Ambulatory navigation/mobility: Sub-group D (0.2–125 lux)	4	66	56	Bainbridge, Russell	D	RR 1.15	CI (0.84, 1.58)	4.30	30%	0.85	0.39
**7**	FST (red light) measurement of log10(cd.s/m^2^)	2	32	24	Bainbridge, Russell	C	MD 0.89	CI (−0.6, 1.84)	5.86	83%	1.84	0.07
**8**	FST (red light) measurement of log10(cd.s/m^2^)	2	32	24	Bainbridge, Russell	D	RR 1.89	CI (1.04, 3.41)	1.74	43%	2.10	* 0.04
**9**	FST (blue light) measurement of log10(cd.s/m^2^)	2	32	24	Bainbridge, Russell	C	MD 1.69	CI (1.21, 2.16)	0.32	0%	6.93	* 0.00001
**10**	FST (blue light) measurement of log10(cd.s/m^2^)	2	32	24	Bainbridge, Russell	D	RR 2.01	CI (1.32, 3.06)	0.63	0%	3.23	* 0.001
**11**	Central retinal thickness (CRT) (1 year)	2	27	27	Bainbridge, Jacobson	D	RR 1.15	CI (0.45, 3.00)	0.16	0%	0.30	0.77
**12**	Central retinal thickness (CRT) (3 year)	2	21	21	Bainbridge, Jacobson	D	RR 1.29	CI (0.33, 5.10)	0.23	0%	0.36	0.72

## Supplementary Materials

The following are available online at https://www.mdpi.com/2218-273X/11/5/760/s1, Figure S1: (a) A simple hierarchy of evidence; (b) A list of 23 assays from the 6 selected studies; (c) Each of the 23 assays re-grouped and colour coded to clearly distinguish how the specific assays were to be used in the study, Figure S2: Visual acuity logMAR, with a random effects model and summary statistic for dichotomous data showed, Table S1: PICOS results. The PICOS search terms, keywords, MeSH terms, search strings and Boolean operators were used and identified in Materials & Method (using Ovid Database), additionally de-fined Appendix A.1, Appendix A.2 and Appendix A.3, Table S2: All mean difference (MD) values for all visual acuity logMAR changes across all six (6) papers. All data was retrieved and analysed by two independent authors, Table S3: All ambulatory navigation/mobility across all six (6) papers. All data was retrieved and analysed by two independent authors, Table S4: (a) MLMT (Russell et al. 2017) and (b) derived data from FDA (BLA No. 125610).

## Appendix A

### Appendix A.1. Ovid Search Results in MEDLINE and EMBASE

The overall aim of this study was to identify a systematic review and meta-analyses of interventional clinical trial studies for gene therapies for IRDs. The aims of the study were to identify, extract, analyse and critique outcomes for gene therapy treatments from a relevant and specific population, in particular:

(i) identify, search and collate the available research data from IRD patients for gene therapy treatment;

(ii) extract and assess the relevant data from an IRD population and prepare a meta-analysis of the available outcomes and potential impact of the research reporting;

(iii) analyse and critique relevant outcomes from key IRD studies.

This systematic review and meta-analyses were performed for interventional clinical trial outcomes for approved gene therapies for IRDs. The systematic review used a structured search approach with a PICOS process (*Population, Intervention, Comparison, Outcomes* and *Study*). The specific research question was to search interventional clinical trials (*the study design*) for published gene therapies (*the intervention*) for IRD patients (*the population*), for the purpose of improving the disorder (*the outcomes*), given there was little or no treatment available (*the comparison*).

The search used the Ovid platform to search the MEDLINE, EMBASE and Cochrane databases. The PICOS search terms and search strings included 36 words and phrases using MESH terms and Boolean operators (within this Appendix A.1), and subsequently reported in Appendix A.2.

**Database: Ovid MEDLINE(R) <1946 to June Week 5 2020>**

Search Strategy:

1. retinitis pigmentosa.mp. [mp = title, abstract, original title, name of substance word, subject heading word, floating sub-heading word, keyword heading word, organism supplementary concept word, protocol supplementary concept word, rare disease supplementary concept word, unique identifier, synonyms] (9772)
2. leber* congenital amaurosis.mp. [mp = title, abstract, original title, name of substance word, subject heading word, floating sub-heading word, keyword heading word, organism supplementary concept word, protocol supplementary concept word, rare disease supplementary concept word, unique identifier, synonyms] (1147)
3. inherited retinal disease.mp. [mp = title, abstract, original title, name of substance word, subject heading word, floating sub-heading word, keyword heading word, organism supplementary concept word, protocol supplementary concept word, rare disease supplementary concept word, unique identifier, synonyms] (150)
4. inherited retinal disorder.mp. [mp = title, abstract, original title, name of substance word, subject heading word, floating sub-heading word, keyword heading word, organism supplementary concept word, protocol supplementary concept word, rare disease supplementary concept word, unique identifier, synonyms] (26)
5. X-linked retinitis pigmentosa.mp. [mp = title, abstract, original title, name of substance word, subject heading word, floating sub-heading word, keyword heading word, organism supplementary concept word, protocol supplementary concept word, rare disease supplementary concept word, unique identifier, synonyms] (311)
6. blindness.mp. [mp = title, abstract, original title, name of substance word, subject heading word, floating sub-heading word, keyword heading word, organism supplementary concept word, protocol supplementary concept word, rare disease supplementary concept word, unique identifier, synonyms] (37,460)
7. rpe65.mp. [mp = title, abstract, original title, name of substance word, subject heading word, floating sub-heading word, keyword heading word, organism supplementary concept word, protocol supplementary concept word, rare disease supplementary concept word, unique identifier, synonyms] (720)
8. exp Eye Diseases, Hereditary/(50,296)
9. **1 or 2 or 3 or 4 or 5 or 6 or 7 or 8 (85,822)**
10. gene therapy.mp. [mp = title, abstract, original title, name of substance word, subject heading word, floating sub-heading word, keyword heading word, organism supplementary concept word, protocol supplementary concept word, rare disease supplementary concept word, unique identifier, synonyms] (41,640)
11. gene replacement.mp. [mp = title, abstract, original title, name of substance word, subject heading word, floating sub-heading word, keyword heading word, organism supplementary concept word, protocol supplementary concept word, rare disease supplementary concept word, unique identifier, synonyms] (2273)
12. recombinant gene.mp. [mp = title, abstract, original title, name of substance word, subject heading word, floating sub-heading word, keyword heading word, organism supplementary concept word, protocol supplementary concept word, rare disease supplementary concept word, unique identifier, synonyms] (733)
13. gene delivery.mp. [mp = title, abstract, original title, name of substance word, subject heading word, floating sub-heading word, keyword heading word, organism supplementary concept word, protocol supplementary concept word, rare disease supplementary concept word, unique identifier, synonyms] (15,306)
14. adeno-associated virus.mp. [mp = title, abstract, original title, name of substance word, subject heading word, floating sub-heading word, keyword heading word, organism supplementary concept word, protocol supplementary concept word, rare disease supplementary concept word, unique identifier, synonyms] (7095)
15. AAV.mp. [mp = title, abstract, original title, name of substance word, subject heading word, floating sub-heading word, keyword heading word, organism supplementary concept word, protocol supplementary concept word, rare disease supplementary concept word, unique identifier, synonyms] (6906)
16. viral delivery.mp. [mp = title, abstract, original title, name of substance word, subject heading word, floating sub-heading word, keyword heading word, organism supplementary concept word, protocol supplementary concept word, rare disease supplementary concept word, unique identifier, synonyms] (630)
17. exp Genetic Therapy/(48,858)
18. **10 or 11 or 12 or 13 or 14 or 15 or 16 or 17 (80,450)**
19. visual acuity.mp. [mp = title, abstract, original title, name of substance word, subject heading word, floating sub-heading word, keyword heading word, organism supplementary concept word, protocol supplementary concept word, rare disease supplementary concept word, unique identifier, synonyms] (90,996)
20. best-corrected visual acuity.mp. [mp = title, abstract, original title, name of substance word, subject heading word, floating sub-heading word, keyword heading word, organism supplementary concept word, protocol supplementary concept word, rare disease supplementary concept word, unique identifier, synonyms] (10,388)
21. standard logarithm of the minimum angle of resolution.mp. [mp = title, abstract, original title, name of substance word, subject heading word, floating sub-heading word, keyword heading word, organism supplementary concept word, protocol supplementary concept word, rare disease supplementary concept word, unique identifier, synonyms] (0)
22. visual field.mp. [mp = title, abstract, original title, name of substance word, subject heading word, floating sub-heading word, keyword heading word, organism supplementary concept word, protocol supplementary concept word, rare disease supplementary concept word, unique identifier, synonyms] (27,503)
23. visual perception.mp. [mp = title, abstract, original title, name of substance word, subject heading word, floating sub-heading word, keyword heading word, organism supplementary concept word, protocol supplementary concept word, rare disease supplementary concept word, unique identifier, synonyms] (64,654)
24. electroretinogram.mp. [mp = title, abstract, original title, name of substance word, subject heading word, floating sub-heading word, keyword heading word, organism supplementary concept word, protocol supplementary concept word, rare disease supplementary concept word, unique identifier, synonyms] (6127)
25. Goldmann visual fields.mp. [mp = title, abstract, original title, name of substance word, subject heading word, floating sub-heading word, keyword heading word, organism supplementary concept word, protocol supplementary concept word, rare disease supplementary concept word, unique identifier, synonyms] (91)
26. microperimetry.mp. [mp = title, abstract, original title, name of substance word, subject heading word, floating sub-heading word, keyword heading word, organism supplementary concept word, protocol supplementary concept word, rare disease supplementary concept word, unique identifier, synonyms] (828)
27. fundus photography.mp. [mp = title, abstract, original title, name of substance word, subject heading word, floating sub-heading word, keyword heading word, organism supplementary concept word, protocol supplementary concept word, rare disease supplementary concept word, unique identifier, synonyms] (2908)
28. nystagmus testing.mp. [mp = title, abstract, original title, name of substance word, subject heading word, floating sub-heading word, keyword heading word, organism supplementary concept word, protocol supplementary concept word, rare disease supplementary concept word, unique identifier, synonyms] (18)
29. central retinal thickness.mp. [mp = title, abstract, original title, name of substance word, subject heading word, floating sub-heading word, keyword heading word, organism supplementary concept word, protocol supplementary concept word, rare disease supplementary concept word, unique identifier, synonyms] (1088)
30. optical coherence tomography.mp. [mp = title, abstract, original title, name of substance word, subject heading word, floating sub-heading word, keyword heading word, organism supplementary concept word, protocol supplementary concept word, rare disease supplementary concept word, unique identifier, synonyms] (26,996)
31. pupillary light reflex response.mp. [mp = title, abstract, original title, name of substance word, subject heading word, floating sub-heading word, keyword heading word, organism supplementary concept word, protocol supplementary concept word, rare disease supplementary concept word, unique identifier, synonyms] (10)
32. full-field light sensitivity threshold.mp. [mp = title, abstract, original title, name of substance word, subject heading word, floating sub-heading word, keyword heading word, organism supplementary concept word, protocol supplementary concept word, rare disease supplementary concept word, unique identifier, synonyms] (2)
33. exp Diagnostic Techniques, Ophthalmological/(169,167)
34. **19 or 20 or 21 or 22 or 23 or 24 or 25 or 26 or 27 or 28 or 29 or 30 or 31 or 32 or 33 (267,300)**
35. clinical trial.mp. [mp = title, abstract, original title, name of substance word, subject heading word, floating sub-heading word, keyword heading word, organism supplementary concept word, protocol supplementary concept word, rare disease supplementary concept word, unique identifier, synonyms] (684,554)
36. randomised clinical trial.mp. [mp = title, abstract, original title, name of substance word, subject heading word, floating sub-heading word, keyword heading word, organism supplementary concept word, protocol supplementary concept word, rare disease supplementary concept word, unique identifier, synonyms] (2676)
37. non-randomised.mp. [mp = title, abstract, original title, name of substance word, subject heading word, floating sub-heading word, keyword heading word, organism supplementary concept word, protocol supplementary concept word, rare disease supplementary concept word, unique identifier, synonyms] (3207)
38. rct.mp. [mp = title, abstract, original title, name of substance word, subject heading word, floating sub-heading word, keyword heading word, organism supplementary concept word, protocol supplementary concept word, rare disease supplementary concept word, unique identifier, synonyms] (18,015)
39. clinical trial/(523,108)
40. **35 or 36 or 37 or 38 or 39 (702,125)**
41. **9 and 18 and 34 and 40 (58)**

**Database: Embase <1980 to 2020 Week 28 >**

Search Strategy:

1. retinitis pigmentosa.mp. [mp = title, abstract, heading word, drug trade name, original title, device manufacturer, drug manufacturer, device trade name, keyword, floating subheading word, candidate term word] (12,771)
2. leber* congenital amaurosis.mp. [mp = title, abstract, heading word, drug trade name, original title, device manufacturer, drug manufacturer, device trade name, keyword, floating subheading word, candidate term word] (2206)
3. inherited retinal disease.mp. [mp = title, abstract, heading word, drug trade name, original title, device manufacturer, drug manufacturer, device trade name, keyword, floating subheading word, candidate term word] (365)
4. inherited retinal disorder.mp. [mp = title, abstract, heading word, drug trade name, original title, device manufacturer, drug manufacturer, device trade name, keyword, floating subheading word, candidate term word] (49)
5. X-linked retinitis pigmentosa.mp. [mp = title, abstract, heading word, drug trade name, original title, device manufacturer, drug manufacturer, device trade name, keyword, floating subheading word, candidate term word] (435)
6. blindness.mp. [mp = title, abstract, heading word, drug trade name, original title, device manufacturer, drug manufacturer, device trade name, keyword, floating subheading word, candidate term word] (56,507)
7. rpe65.mp. [mp = title, abstract, heading word, drug trade name, original title, device manufacturer, drug manufacturer, device trade name, keyword, floating subheading word, candidate term word] (1412)
8. exp eye disease/(860,410)
9. **1 or 2 or 3 or 4 or 5 or 6 or 7 or 8 (867,550)**
10. gene therapy.mp. [mp = title, abstract, heading word, drug trade name, original title, device manufacturer, drug manufacturer, device trade name, keyword, floating subheading word, candidate term word] (98,379)
11. gene replacement.mp. [mp = title, abstract, heading word, drug trade name, original title, device manufacturer, drug manufacturer, device trade name, keyword, floating subheading word, candidate term word] (3771)
12. recombinant gene.mp. [mp = title, abstract, heading word, drug trade name, original title, device manufacturer, drug manufacturer, device trade name, keyword, floating subheading word, candidate term word] (2354)
13. gene delivery.mp. [mp = title, abstract, heading word, drug trade name, original title, device manufacturer, drug manufacturer, device trade name, keyword, floating subheading word, candidate term word] (45,914)
14. adeno-associated virus.mp. [mp = title, abstract, heading word, drug trade name, original title, device manufacturer, drug manufacturer, device trade name, keyword, floating subheading word, candidate term word] (16,154)
15. AAV.mp. [mp = title, abstract, heading word, drug trade name, original title, device manufacturer, drug manufacturer, device trade name, keyword, floating subheading word, candidate term word] (15,822)
16. viral delivery.mp. [mp = title, abstract, heading word, drug trade name, original title, device manufacturer, drug manufacturer, device trade name, keyword, floating subheading word, candidate term word] (1057)
17. gene therapy/(60,417)
18. **10 or 11 or 12 or 13 or 14 or 15 or 16 or 17 (138,881)**
19. visual acuity.mp. [mp = title, abstract, heading word, drug trade name, original title, device manufacturer, drug manufacturer, device trade name, keyword, floating subheading word, candidate term word] (133,479)
20. best-corrected visual acuity.mp. [mp = title, abstract, heading word, drug trade name, original title, device manufacturer, drug manufacturer, device trade name, keyword, floating subheading word, candidate term word] (22,009)
21. standard logarithm of the minimum angle of resolution.mp. [mp = title, abstract, heading word, drug trade name, original title, device manufacturer, drug manufacturer, device trade name, keyword, floating subheading word, candidate term word] (3)
22. visual field.mp. [mp = title, abstract, heading word, drug trade name, original title, device manufacturer, drug manufacturer, device trade name, keyword, floating subheading word, candidate term word] (50,559)
23. visual perception.mp. [mp = title, abstract, heading word, drug trade name, original title, device manufacturer, drug manufacturer, device trade name, keyword, floating subheading word, candidate term word] (8084)
24. electroretinogram.mp. [mp = title, abstract, heading word, drug trade name, original title, device manufacturer, drug manufacturer, device trade name, keyword, floating subheading word, candidate term word] (12,950)
25. Goldmann visual fields.mp. [mp = title, abstract, heading word, drug trade name, original title, device manufacturer, drug manufacturer, device trade name, keyword, floating subheading word, candidate term word] (144)
26. microperimetry.mp. [mp = title, abstract, heading word, drug trade name, original title, device manufacturer, drug manufacturer, device trade name, keyword, floating subheading word, candidate term word] (1575)
27. fundus photography.mp. [mp = title, abstract, heading word, drug trade name, original title, device manufacturer, drug manufacturer, device trade name, keyword, floating subheading word, candidate term word] (4794)
28. nystagmus testing.mp. [mp = title, abstract, heading word, drug trade name, original title, device manufacturer, drug manufacturer, device trade name, keyword, floating subheading word, candidate term word] (24)
29. central retinal thickness.mp. [mp = title, abstract, heading word, drug trade name, original title, device manufacturer, drug manufacturer, device trade name, keyword, floating subheading word, candidate term word] (2714)
30. optical coherence tomography.mp. [mp = title, abstract, heading word, drug trade name, original title, device manufacturer, drug manufacturer, device trade name, keyword, floating subheading word, candidate term word] (66,975)
31. pupillary light reflex response.mp. [mp = title, abstract, heading word, drug trade name, original title, device manufacturer, drug manufacturer, device trade name, keyword, floating subheading word, candidate term word] (13)
32. full-field light sensitivity threshold.mp. [mp = title, abstract, heading word, drug trade name, original title, device manufacturer, drug manufacturer, device trade name, keyword, floating subheading word, candidate term word] (10)
33. exp visual system examination/or exp visual system function/or exp visual system parameters/or exp visual threshold/(472,418)
34. **19 or 20 or 21 or 22 or 23 or 24 or 25 or 26 or 27 or 28 or 29 or 30 or 31 or 32 or 33 (520,911)**
35. clinical trial.mp. [mp = title, abstract, heading word, drug trade name, original title, device manufacturer, drug manufacturer, device trade name, keyword, floating subheading word, candidate term word] (1,537,834)
36. randomised clinical trial.mp. [mp = title, abstract, heading word, drug trade name, original title, device manufacturer, drug manufacturer, device trade name, keyword, floating subheading word, candidate term word] (4255)
37. non-randomised.mp. [mp = title, abstract, heading word, drug trade name, original title, device manufacturer, drug manufacturer, device trade name, keyword, floating subheading word, candidate term word] (5299)
38. rct.mp. [mp = title, abstract, heading word, drug trade name, original title, device manufacturer, drug manufacturer, device trade name, keyword, floating subheading word, candidate term word] (39,586)
39. clinical trial/(966,569)
40. **35 or 36 or 37 or 38 or 39 (1,567,775)**
41. **exp controlled clinical trial/(792,290)**
42. **9 and 18 and 34 and 40 and 41 (55)**

### Appendix A.2. Results of Searches, Papers and Assessment of Data Using Ovid in MEDLINE and EMBASE

**No. References (listed alphabetically)**

1. Aleman T.S., Serrano L., Han G.K., Pearson D.J., McCague S., Marshall K.A., Chung D.C., Liu E., Morgan J.I.W., Bennett J., Maguire A.M. Investigative Ophthalmology and Visual Science. Conference: 2017 Annual Meeting of the Association for Research in Vision and Ophthalmology, ARVO 2017. United States. 58 (8) (no pagination), 2017. Date of Publication: June 2017 AAV2-hCHM subretinal delivery to the macula in choroideremia: preliminary six-month safety results of an ongoing phase I/II gene therapy trial.
2. Anonymous Neuropediatrics. Conference: 47th Annual Meeting of the Societe Europeenne de Neurologie Pediatrique, SENP 2019. France. 50 (Supplement 1) (no pagination), 2019. Date of Publication: March 2019. Abstracts of the 47th Annual Meeting of the SENP (Societe Europeenne de Neurologie Pediatrique).
3. Ashtari M; Cyckowski LL; Monroe JF; Marshall KA; Chung DC; Auricchio A; Simonelli F; Leroy BP; Maguire AM; Shindler KS; Bennett J. Journal of Clinical Investigation. 121(6):2160–8, 2011 Jun. The human visual cortex responds to gene therapy-mediated recovery of retinal function.
4. Ashtari M; Nikonova ES; Marshall KA; Young GJ; Aravand P; Pan W; Ying GS; Willett AE; Mahmoudian M; Maguire AM; Bennett J. Ophthalmology. 124(6):873–883, 2017 06. The Role of the Human Visual Cortex in Assessment of the Long-Term Durability of Retinal Gene Therapy in Follow-on RPE65 Clinical Trial Patients.
5. Ashtari M., Nikonova E.S., Marshall K.A., Young G.J., Aravand P., Pan W., Ying G.-S., Willett A.E., Mahmoudian M., Maguire A.M., Bennett J. Molecular Therapy. Conference: 20th Annual Meeting of the American Society of Gene and Cell Therapy, ASGCT 2017. United States. 25 (5 Supplement 1) (pp 138), 2017. Date of Publication: May 2017 Does a one-time retinal gene therapy last long: A question answered by the brain.
6. Audo I.S., Weleber R.G., Stout T., Lauer A.K., Pennesi M.E., Mohand-Said S., Barale P.-O., Buggage R., Wilson D.J., Sahel J.A. Investigative Ophthalmology and Visual Science. Conference: 2015 Annual Meeting of the Association for Research in Vision and Ophthalmology, ARVO 2015. United States. 56 (7) (pp 3819), 2015. Date of Publication: June 2015 Early findings in a phase I/IIa clinical program for stargardt disease (STGD1, MIM #248200).
7. Bainbridge JW; Mehat MS; Sundaram V; Robbie SJ; Barker SE; Ripamonti C; Georgiadis A; Mowat FM; Beattie SG; Gardner PJ; Feathers KL; Luong VA; Yzer S; Balaggan K; Viswanathan A; de Ravel TJ; Casteels I; Holder GE; Tyler N; Fitzke FW; Weleber RG; Nardini M; Moore AT; Thompson DA; Petersen-Jones SM; Michaelides M; van den Born LI; Stockman A; Smith AJ; Rubin G; Ali RR. New England Journal of Medicine. 372(20):1887–97, 2015 May 14 Long-term effect of gene therapy on Leber’s congenital amaurosis.
8. Bainbridge JW; Smith AJ; Barker SS; Robbie S; Henderson R; Balaggan K; Viswanathan A; Holder GE; Stockman A; Tyler N; Petersen-Jones S; Bhattacharya SS; Thrasher AJ; Fitzke FW; Carter BJ; Rubin GS; Moore AT; Ali RR. New England Journal of Medicine. 358(21):2231–9, 2008 May 22. Effect of gene therapy on visual function in Leber’s congenital amaurosis.
9. Banin E; Bandah-Rozenfeld D; Obolensky A; Cideciyan AV; Aleman TS; Marks-Ohana D; Sela M; Boye S; Sumaroka A; Roman AJ; Schwartz SB; Hauswirth WW; Jacobson SG; Hemo I; Sharon D. Human Gene Therapy. 21(12):1749–57, 2010 Dec Molecular anthropology meets genetic medicine to treat blindness in the North African Jewish population: human gene therapy initiated in Israel.
10. Beltran WA; Cideciyan AV; Boye SE; Ye GJ; Iwabe S; Dufour VL; Marinho LF; Swider M; Kosyk MS; Sha J; Boye SL; Peterson JJ; Witherspoon CD; Alexander JJ; Ying GS; Shearman MS; Chulay JD; Hauswirth WW; Gamlin PD; Jacobson SG; Aguirre GD. Molecular Therapy: The Journal of the American Society of Gene Therapy. 25(8):1866–1880, 2017 08 02 Optimization of Retinal Gene Therapy for X-Linked Retinitis Pigmentosa Due to RPGR Mutations.
11. Benjaminy S; Macdonald I; Bubela T. Genetics in Medicine. 16(5):379–85, 2014 May. Is a cure in my sight? Multi-stakeholder perspectives on phase I choroideremia gene transfer clinical trials.
12. Bennett J; Wellman J; Marshall KA; McCague S; Ashtari M; DiStefano-Pappas J; Elci OU; Chung DC; Sun J; Wright JF; Cross DR; Aravand P; Cyckowski LL; Bennicelli JL; Mingozzi F; Auricchio A; Pierce EA; Ruggiero J; Leroy BP; Simonelli F; High KA; Maguire AM.Lancet. 388(10045):661–72, 2016 Aug 13. Safety and durability of effect of contralateral-eye administration of AAV2 gene therapy in patients with childhood-onset blindness caused by RPE65 mutations: a follow-on phase 1 trial.
13. Bennett L.D., Pennesi M.E., Niimi J., Wilson D.J., Erker L., Parker M., Heckenlively J.R., Branham K.E., Birch D.G. Investigative Ophthalmology and Visual Science. Conference: 2015 Annual Meeting of the Association for Research in Vision and Ophthalmology, ARVO 2015. United States. 56 (7) (pp 3834), 2015. Date of Publication: June 2015 Outer segment thickness rather than total retina thickness predicts macular function in X-Linked Retinoschisis (XLRS).
14. Bouquet C; Vignal Clermont C; Galy A; Fitoussi S; Blouin L; Munk MR; Valero S; Meunier S; Katz B; Sahel JA; Thomasson N. JAMA Ophthalmology. 137(4):399–406, 2019 04 01. Immune Response and Intraocular Inflammation in Patients With Leber Hereditary Optic Neuropathy Treated With Intravitreal Injection of Recombinant Adeno-Associated Virus 2 Carrying the ND4 Gene: A Secondary Analysis of a Phase 1/2 Clinical Trial.
15. Bouquet C., Douar A., Chavas J., Pruneau D., Cancian C., Thomasson N. Human Gene Therapy. Conference: 25th Anniversary Congress of the European Society of Gene and Cell Therapy, ESGCT 2017. Germany. 28 (12) (pp A80-A81), 2017. Date of Publication: 2017 Ocular tolerability of AAV2.7m8-ChrimsonR-tdTomato (GS030-DP) gene therapy product on blind rd1 mice injected intravitreously and exposed to 595 nm LED light.
16. Bouquet C., Vignal Clermont C., Galy A., Fitoussi S., Blouin L., Munk M.R., Valero S., Meunier S., Katz B., Sahel J.A., Thomasson N. JAMA Ophthalmology. 137 (4) (pp 399–406), 2019. Date of Publication: April 2019 Immune Response and Intraocular Inflammation in Patients with Leber Hereditary Optic Neuropathy Treated with Intravitreal Injection of Recombinant Adeno-Associated Virus 2 Carrying the ND4 Gene: A Secondary Analysis of a Phase 1/2 Clinical Trial.
17. Caruso RC; Nussenblatt RB; Csaky KG; Valle D; Kaiser-Kupfer MI. Archives of Ophthalmology. 119(5):667–9, 2001 May. Assessment of visual function in patients with gyrate atrophy who are considered candidates for gene replacement.
18. Cehajic-Kapetanovic J; Xue K; Martinez-Fernandez de la Camara C; Nanda A; Davies A; Wood LJ; Salvetti AP; Fischer MD; Aylward JW; Barnard AR; Jolly JK; Luo E; Lujan BJ; Ong T; Girach A; Black GCM; Gregori NZ; Davis JL; Rosa PR; Lotery AJ; Lam BL; Stanga PE; MacLaren RE. Nature Medicine. 26(3):354–359, 2020 03. Initial results from a first-in-human gene therapy trial on X-linked retinitis pigmentosa caused by mutations in RPGR. EXCLUDED STUDY
19. Chacon-Camacho OF; Zenteno JC. Gaceta Medica de Mexico. 153(2):276–278, 2017 Mar–Apr [Gene therapy for vision restoration in patients with Leber congenital amaurosis (LCA) due to RPE65 gene mutations: beginning the phase IV trial]. [Spanish] Terapia genica para la restauracion de la vision en pacientes con amaurosis congenita de Leber (LCA) por mutacion en el gen RPE65: el inicio de la fase IV.
20. Chevez-Barrios P., Chintagumpala M., Mieler W., Paysse E., Boniuk M., Kozinetz C., Hurwitz M.Y., Hurwitz R.L. Journal of Clinical Oncology. 23 (31) (pp 7927–7935), 2005. Date of Publication: 2005 Response of retinoblastoma with vitreous tumor seeding to adenovirus-mediated delivery of thymidine kinase followed by ganciclovir.
21. Chiocca E.A., Smith K.M., McKinney B., Palmer C.A., Rosenfeld S., Lillehei K., Hamilton A., DeMasters B.K., Judy K., Kirn D. Molecular Therapy. 16 (3) (pp 618–626), 2008. Date of Publication: March 2008 A phase I trial of ad.hIFN-beta gene therapy for glioma.
22. Cideciyan AV; Aguirre GK; Jacobson SG; Butt OH; Schwartz SB; Swider M; Roman AJ; Sadigh S; Hauswirth WW. Investigative Ophthalmology & Visual Science. 56(1):526–37, 2014 Dec 23. Pseudo-fovea formation after gene therapy for RPE65-LCA.
23. Cideciyan AV; Charng J; Roman AJ; Sheplock R; Garafalo AV; Heon E; Jacobson SG. Investigative Ophthalmology & Visual Science. 59(11):4558–4566, 2018 09 04 Progression in X-linked Retinitis Pigmentosa Due to ORF15-RPGR Mutations: Assessment of Localized Vision Changes Over 2 Years.
24. Cideciyan AV; Hauswirth WW; Aleman TS; Kaushal S; Schwartz SB; Boye SL; Windsor EA; Conlon TJ; Sumaroka A; Pang JJ; Roman AJ; Byrne BJ; Jacobson SG. Human Gene Therapy. 20(9):999–1004, 2009 Sep Human RPE65 gene therapy for Leber congenital amaurosis: persistence of early visual improvements and safety at 1 year.
25. Comer G.M., Ciulla T.A., Criswell M.H., Tolentino M. Drugs and Aging. 21 (15) (pp 967–992), 2004. Date of Publication: 2004 Current and future treatment options for nonexudative and exudative age-related macular degeneration.
26. Conlon TJ; Deng WT; Erger K; Cossette T; Pang JJ; Ryals R; Clement N; Cleaver B; McDoom I; Boye SE; Peden MC; Sherwood MB; Abernathy CR; Alkuraya FS; Boye SL; Hauswirth WW. Human Gene Therapy. 24(1):23–8, 2013 Mar Preclinical potency and safety studies of an AAV2-mediated gene therapy vector for the treatment of MERTK associated retinitis pigmentosa.
27. Constable I.J., Lai C.-M., Magno A.L., French M.A., Barone S.B., Schwartz S.D., Blumenkranz M.S., Degli-Esposti M.A., Rakoczy E.P. American Journal of Ophthalmology. 177 (pp 150–158), 2017. Date of Publication: 01 May 2017 Gene Therapy in Neovascular Age-related Macular Degeneration: Three-Year Follow-up of a Phase 1 Randomized Dose Escalation Trial.
28. Constable I.J., Pierce C.M., Lai C.-M., Magno A.L., Degli-Esposti M.A., French M.A., McAllister I.L., Butler S., Barone S.B., Schwartz S.D., Blumenkranz M.S., Rakoczy E.P. EBioMedicine. 14 (pp 168–175), 2016. Date of Publication: 01 Dec 2016 Phase 2a Randomized Clinical Trial: Safety and Post Hoc Analysis of Subretinal rAAV.sFLT-1 for Wet Age-related Macular Degeneration.
29. Couto L.B., Buchlis G., Farjo R., High K. Investigative Ophthalmology and Visual Science. Conference: 2016 Annual Meeting of the Association for Research in Vision and Ophthalmology, ARVO 2016. United States. 57 (12) (pp 759), 2016. Date of Publication: September 2016 Potency assay for AAV vector encoding retinal pigment epithelial 65 protein.
30. Dimopoulos IS; Hoang SC; Radziwon A; Binczyk NM; Seabra MC; MacLaren RE; Somani R; Tennant MTS; MacDonald IM. American Journal of Ophthalmology. 193:130–142, 2018 09 Two-Year Results After AAV2-Mediated Gene Therapy for Choroideremia: The Alberta Experience.
31. Drack A.V., Bennett J., Russell S., High K.A., Yu Z.-F., Tillman A., Chung D., Reape K.Z., Ciulla T., Maguire A. Journal of AAPOS. Conference: The 45th Annual Meeting of the American Association for Pediatric Ophthalmology and Strabismus. United States. 23 (4) (pp e7), 2019. Date of Publication: August 2019 How long does gene therapy last? 4-year follow-up of phase 3 voretigene neparvovec trial in RPE65-associated LCA/inherited retinal disease.
32. Dufier JL. Bulletin de l Academie Nationale de Medecine. 187(9):1685–92; discussion 1692–4, 2003 (Early therapeutic trials for retinitis pigmentosa). (Review) (17 Refs) (French) La retinopathie pigmentaire a la recherche d’une approche therapeutique.
33. Feuer WJ; Schiffman JC; Davis JL; Porciatti V; Gonzalez P; Koilkonda RD; Yuan H; Lalwani A; Lam BL; Guy J. Ophthalmology. 123(3):558–70, 2016 Mar. Gene Therapy for Leber Hereditary Optic Neuropathy: Initial Results.
34. Fischer M.D., McClements M.E., De La Camar C.M.-F., Bellingrath J.-S., Dauletbekov D., Ramsden S.C., Hickey D.G., Barnard A.R., MacLaren R.E. Investigative Ophthalmology and Visual Science. Conference: 2017 Annual Meeting of the Association for Research in Vision and Ophthalmology, ARVO 2017. United States. 58 (8) (no pagination), 2017. Date of Publication: June 2017 Codon optimized RPGR leads to improved stability and rescue with AAV8 gene therapy in X-linked retinitis pigmentosa.
35. Fischer M.D., Michalakis S., Wilhelm B., Zobor D., Muehlfriedel R., Kohl S., Weisschuh N., Ochakovski G.A., Klein R., Schoen C., Sothilingam V., Garcia-Garrido M., Kuehlewein L., Kahle N., Werner A., Dauletbekov D., Paquet-Durand F., Tsang S., Martus P., Peters T., Seeliger M., Bartz-Schmidt K.U., Ueffing M., Zrenner E., Biel M., Wissinger B. JAMA Ophthalmology. (no pagination), 2020. Date of Publication: 2020. Safety and Vision Outcomes of Subretinal Gene Therapy Targeting Cone Photoreceptors in Achromatopsia: A Nonrandomized Controlled Trial.
36. Fischer M.D., Ochakovski G.A., Beier B., Seitz I.P., Vaheb Y., Kortuem C., Reichel F.F.L., Kuehlewein L., Kahle N.A., Peters T., Girach A., Zrenner E., Ueffing M., Maclaren R.E., Bartz-Schmidt K., Wilhelm B. Retina. 40 (1) (pp 160–168), 2020. Date of Publication: 01 Jan 2020 CHANGES in RETINAL SENSITIVITY after GENE THERAPY in CHOROIDEREMIA.
37. Fischer M.D., Ochakovski G.A., Beier B., Seitz I.P., Vaheb Y., Kortuem C., Reichel F.F.L., Kuehlewein L., Kahle N.A., Peters T., Girach A., Zrenner E., Ueffing M., MacLaren R.E., Bartz-Schmidt K.U., Wilhelm B. JAMA Ophthalmology. 137 (11) (pp. 1247–1254), 2019. Date of Publication: November 2019 Efficacy and Safety of Retinal Gene Therapy Using Adeno-Associated Virus Vector for Patients with Choroideremia: A Randomized Clinical Trial.
38. Fischer M.D., Wilhelm B., Zrenner E., Ueffing M., Wissinger B., Biel M., Bartz-Schmidt K.U. Ophthalmologica. Conference: 16th Euretina Congress. Denmark. 236 (Supplement 1) (pp 30), 2016. Date of Publication: September 2016 Safe delivery of raav8.CNGA3 in patients with achromatopsia.
39. Ghazi NG; Abboud EB; Nowilaty SR; Alkuraya H; Alhommadi A; Cai H; Hou R; Deng WT; Boye SL; Almaghamsi A; Al Saikhan F; Al-Dhibi H; Birch D; Chung C; Colak D; LaVail MM; Vollrath D; Erger K; Wang W; Conlon T; Zhang K; Hauswirth W; Alkuraya FS. Human Genetics. 135(3):327–43, 2016 Mar. Treatment of retinitis pigmentosa due to MERTK mutations by ocular subretinal injection of adeno-associated virus gene vector: results of a phase I trial.
40. Guy J; Feuer WJ; Davis JL; Porciatti V; Gonzalez PJ; Koilkonda RD; Yuan H; Hauswirth WW; Lam BL.Ophthalmology. 124(11):1621–1634, 2017 11 Gene Therapy for Leber Hereditary Optic Neuropathy: Low- and Medium-Dose Visual Results.
41. Hassall M.M., McClements M.E., Barnard A.R., Aslam S.A., MacLaren R.E. Investigative Ophthalmology and Visual Science. Conference: 2017 Annual Meeting of the Association for Research in Vision and Ophthalmology, ARVO 2017. United States. 58 (8) (no pagination), 2017. Date of Publication: June 2017. Cone opsins and Crx are gene therapy candidates for the revival of cone photoreceptors in an RP mouse model.
42. Hauswirth WW; Aleman TS; Kaushal S; Cideciyan AV; Schwartz SB; Wang L; Conlon TJ; Boye SL; Flotte TR; Byrne BJ; Jacobson SG. Human Gene Therapy. 19(10):979–90, 2008 Oct Treatment of leber congenital amaurosis due to RPE65 mutations by ocular subretinal injection of adeno-associated virus gene vector: short-term results of a phase I trial.
43. Hernandez C., Simo R. Expert Opinion on Investigational Drugs. 16 (8) (pp 1209–1226), 2007. Date of Publication: August 2007. Strategies for blocking angiogenesis in diabetic retinopathy: From basic science to clinical practice.
44. Huang S.S. Asia-Pacific journal of ophthalmology (Philadelphia, Pa.). 9 (3) (pp 180–185), 2020. Date of Publication: 01 May 2020. Future Vision 2020 and Beyond-5 Critical Trends in Eye Research. Huang S.S.
45. Ikeda Y; Yonemitsu Y; Miyazaki M; Kohno R; Murakami Y; Murata T; Goto Y; Tabata T; Ueda Y; Ono F; Suzuki T; Ageyama N; Terao K; Hasegawa M; Sueishi K; Ishibashi T. Human Gene Therapy. 20(9):943–54, 2009 Sep. Acute toxicity study of a simian immunodeficiency virus-based lentiviral vector for retinal gene transfer in nonhuman primates.
46. Jacobson S.G., Cideciyan A.V., Ratnakaram R., Heon E., Schwartz S.B., Roman A.J., Peden M.C., Aleman T.S., Boye S.L., Sumaroka A., Conlon T.J., Calcedo R., Pang J.-J., Erger K.E., Olivares M.B., Mullins C.L., Swider M., Kaushal S., Feuer W.J., Iannaccone A., Fishman G.A., Stone E.M., Byrne B.J., Hauswirth W.W. Archives of Ophthalmology. 130 (1) (pp 9–24), 2012. Date of Publication: January 2012 Gene therapy for leber congenital amaurosis caused by RPE65 mutations: Safety and efficacy in 15 children and adults followed up to 3 years.
47. Jacobson SG; Cideciyan AV; Ratnakaram R; Heon E; Schwartz SB; Roman AJ; Peden MC; Aleman TS; Boye SL; Sumaroka A; Conlon TJ; Calcedo R; Pang JJ; Erger KE; Olivares MB; Mullins CL; Swider M; Kaushal S; Feuer WJ; Iannaccone A; Fishman GA; Stone EM; Byrne BJ; Hauswirth WW. Archives of Ophthalmology. 130(1):9–24, 2012 Jan. Gene therapy for leber congenital amaurosis caused by RPE65 mutations: safety and efficacy in 15 children and adults followed up to 3 years.
48. Jolly JK; Xue K; Edwards TL; Groppe M; MacLaren RE. Investigative Ophthalmology & Visual Science. 58(12):5575–5583, 2017 10 01. Characterizing the Natural History of Visual Function in Choroideremia Using Microperimetry and Multimodal Retinal Imaging.
49. Kachi S; Ishikawa K; Terasaki H. Nippon Ganka Gakkai Zasshi—Acta Societatis Ophthalmologicae Japonicae. 113(4):479–91, 2009 Apr. [New therapies for age-related macular degeneration]. [Review] [83 refs] [Japanese]
50. Kearns L.S., Staffieri S.E., Ruddle J.B., Hewitt A.W., Mackey D. Clinical and Experimental Ophthalmology. Conference: 49th Annual Scientific Congress of the Royal Australian and New Zealand College of Ophthalmologists. Australia. 45 (Supplement 1) (pp 114), 2017. Date of Publication: October 2017. In pursuit of gene therapy trials and better sight for young males with Leber’s Hereditary Optic Neuropathy (LHON).
51. Koilkonda R; Yu H; Talla V; Porciatti V; Feuer WJ; Hauswirth WW; Chiodo V; Erger KE; Boye SL; Lewin AS; Conlon TJ; Renner L; Neuringer M; Detrisac C; Guy J. Investigative Ophthalmology & Visual Science. 55(12):7739–53, 2014 Oct 23 LHON gene therapy vector prevents visual loss and optic neuropathy induced by G11778A mutant mitochondrial DNA: biodistribution and toxicology profile.
52. Koilkonda RD; Yu H; Chou TH; Feuer WJ; Ruggeri M; Porciatti V; Tse D; Hauswirth WW; Chiodo V; Boye SL; Lewin AS; Neuringer M; Renner L; Guy J. JAMA Ophthalmology. 132(4):409–20, 2014 Apr 01 Safety and effects of the vector for the Leber hereditary optic neuropathy gene therapy clinical trial.
53. Komaromy AM; Varner SE; de Juan E; Acland GM; Aguirre GD. Cell Transplantation. 15(6):511–9, 2006. Application of a new subretinal injection device in the dog.
54. Lai C.-M., Magno A., Pierce C., Samulski R.J., Chalberg T.W., Blumenkranz M.S., Constable I.J., Rakoczy E.P. Journal of Gene Medicine. Conference: 8th Australasian Gene Therapy Society Meeting. Australia. 15 [8,9] (pp 319), 2013. Date of Publication: August-September 2013 Results from a phase I/II clinical trial on anti-vascular endothelial growth factor gene therapy in patients with exudative age-related macular degeneration.
55. Lai C.-M., Magno A.L., Barone S.B., Schwartz S.D., Blumenkranz M.S., Constable I.J., Rakoczy E.P. Molecular Therapy. Conference: 20th Annual Meeting of the American Society of Gene and Cell Therapy, ASGCT 2017. United States. 25 (5 Supplement 1) (pp 315), 2017. Date of Publication: May 2017 Optical coherence tomography profiles, visual acuity and ranibizumab usage during 3-year follow-up in a rAAV.sFLT-1 gene therapy clinical trial for wet age-related macular degeneration.
56. Lam BL; Davis JL; Gregori NZ; MacLaren RE; Girach A; Verriotto JD; Rodriguez B; Rosa PR; Zhang X; Feuer WJ. American Journal of Ophthalmology. 197:65–73, 2019 01. EXCLUDED STUDY Choroideremia Gene Therapy Phase 2 Clinical Trial: 24-Month Results.
57. Lam BL; Feuer WJ; Abukhalil F; Porciatti V; Hauswirth WW; Guy J. Archives of Ophthalmology. 128(9):1129–35, 2010 Sep Leber hereditary optic neuropathy gene therapy clinical trial recruitment: year 1.
58. Lam BL; Feuer WJ; Schiffman JC; Porciatti V; Vandenbroucke R; Rosa PR; Gregori G; Guy J. JAMA Ophthalmology. 132(4):428–36, 2014 Apr 01. Trial end points and natural history in patients with G11778A Leber hereditary optic neuropathy: preparation for gene therapy clinical trial.
59. Lambertus S; Bax NM; Fakin A; Groenewoud JM; Klevering BJ; Moore AT; Michaelides M; Webster AR; van der Wilt GJ; Hoyng CB. PLoS ONE [Electronic Resource]. 12(3):e0174020, 2017. Highly sensitive measurements of disease progression in rare disorders: Developing and validating a multimodal model of retinal degeneration in Stargardt disease.
60. Le Meur G; Lebranchu P; Billaud F; Adjali O; Schmitt S; Bezieau S; Pereon Y; Valabregue R; Ivan C; Darmon C; Moullier P; Rolling F; Weber M. Molecular Therapy: the Journal of the American Society of Gene Therapy. 26(1):256–268, 2018 01 03. Safety and Long-Term Efficacy of AAV4 Gene Therapy in Patients with RPE65 Leber Congenital Amaurosis.
61. Leroy B.P., Maguire A.M., Russell S.R., Yu Z.-F., Wellman J., Bennett J., High K.A. Ophthalmologica. Conference: 16th Euretina Congress. Denmark. 236 (Supplement 1) (pp 2), 2016. Date of Publication: September 2016. Phase 3 efficacy and safety study of voretigene neparvovec (AAV2-HRPE65V2) in subjects with RPE65-mediated inherited retinal dystrophy.
62. Luo X; Cideciyan AV; Iannaccone A; Roman AJ; Ditta LC; Jennings BJ; Yatsenko SA; Sheplock R; Sumaroka A; Swider M; Schwartz SB; Wissinger B; Kohl S; Jacobson SG.PLoS ONE (Electronic Resource). 10(4):e0125700, 2015. Blue cone monochromacy: visual function and efficacy outcome measures for clinical trials.
63. MacLaren R.E., Xue K., Barnard A.R., Patricio M.I., Edwards T.L., Downes S., Lotery A., Black G., Webster A., Jolly J.K., Seabra M.C. Investigative Ophthalmology and Visual Science. Conference: 2018 Annual Meeting of the Association for Research in Vision and Ophthalmology, ARVO 2018. United States. 59 (9) (no pagination), 2018. Date of Publication: July 2018 Retinal gene therapy for choroideremia in a multicenter dose escalation phase I/II clinical trial.
64. MacLaren RE; Groppe M; Barnard AR; Cottriall CL; Tolmachova T; Seymour L; Clark KR; During MJ; Cremers FP; Black GC; Lotery AJ; Downes SM; Webster AR; Seabra MC. Lancet. 383(9923):1129–37, 2014 Mar 29. Retinal gene therapy in patients with choroideremia: initial findings from a phase 1/2 clinical trial.
65. Magno A.L., Lai C.M., Pierce C., Chalberg T.W., Schwartz S., Blumenkranz M.S., French M., Constable I.J., Rakoczy E.P. Journal of Gene Medicine. Conference: 9th Australasian Gene and Cell Therapy Society Meeting. Australia. 17 (8–9) (pp 191), 2015. Date of Publication: August-September 2015 A phase 1 gene therapy trial with subretinal rAAV. sflt-1 for the long-term treatment of wet agerelated macular degeneration: 1-year follow-up.
66. Maguire A.M., Russell S., Wellman J.A., Chung D.C., Yu Z.-F., Tillman A., Wittes J., Pappas J., Elci O., Marshall K.A., McCague S., Reichert H., Davis M., Simonelli F., Leroy B.P., Wright J.F., High K.A., Bennett J. Ophthalmology. 126 (9) (pp 1273–1285), 2019. Date of Publication: September 2019 Efficacy, Safety, and Durability of Voretigene Neparvovec-rzyl in RPE65 Mutation-Associated Inherited Retinal Dystrophy: Results of Phase 1 and 3 Trials.
67. Maguire AM; High KA; Auricchio A; Wright JF; Pierce EA; Testa F; Mingozzi F; Bennicelli JL; Ying GS; Rossi S; Fulton A; Marshall KA; Banfi S; Chung DC; Morgan JI; Hauck B; Zelenaia O; Zhu X; Raffini L; Coppieters F; De Baere E; Shindler KS; Volpe NJ; Surace EM; Acerra C; Lyubarsky A; Redmond TM; Stone E; Sun J; McDonnell JW; Leroy BP; Simonelli F; Bennett J. Lancet. 374(9701):1597–605, 2009 Nov 07 Age-dependent effects of RPE65 gene therapy for Leber’s congenital amaurosis: a phase 1 dose-escalation trial.
68. Maguire AM; Russell S; Wellman JA; Chung DC; Yu ZF; Tillman A; Wittes J; Pappas J; Elci O; Marshall KA; McCague S; Reichert H; Davis M; Simonelli F; Leroy BP; Wright JF; High KA; Bennett J. Efficacy, Safety, and Durability of Voretigene Neparvovec-rzyl in RPE65 Mutation-Associated Inherited Retinal Dystrophy: Results of Phase 1 and 3 Trials. Ophthalmology. 126(9):1273–1285, 2019 09.
69. Maguire AM; Simonelli F; Pierce EA; Pugh EN Jr; Mingozzi F; Bennicelli J; Banfi S; Marshall KA; Testa F; Surace EM; Rossi S; Lyubarsky A; Arruda VR; Konkle B; Stone E; Sun J; Jacobs J; Dell’Osso L; Hertle R; Ma JX; Redmond TM; Zhu X; Hauck B; Zelenaia O; Shindler KS; Maguire MG; Wright JF; Volpe NJ; McDonnell JW; Auricchio A; High KA; Bennett J. New England Journal of Medicine. 358(21):2240–8, 2008 May 22. Safety and efficacy of gene transfer for Leber’s congenital amaurosis.
70. Mihelec M; Pearson RA; Robbie SJ; Buch PK; Azam SA; Bainbridge JW; Smith AJ; Ali RR. Human Gene Therapy. 22(10):1179–90, 2011 Oct Long-term preservation of cones and improvement in visual function following gene therapy in a mouse model of leber congenital amaurosis caused by guanylate cyclase-1 deficiency.
71. Parker M., Weleber R.G., Stout T., Erker L., Audo I.S., Mohand-Said S., Barale P.-O., Buggage R., Sahel J.A., Wilson D.J. Investigative Ophthalmology and Visual Science. Conference: 2015 Annual Meeting of the Association for Research in Vision and Ophthalmology, ARVO 2015. United States. 56 (7) (pp 520), 2015. Date of Publication: June 2015 Foveal detachment in patients undergoing gene therapy for Stargardt disease (STGD1, MIM #248200).
72. Patricio MI; Barnard AR; Xue K; MacLaren RE. Expert Opinion on Biological Therapy. 18(7):807–820, 2018 07. Choroideremia: molecular mechanisms and development of AAV gene therapy. [Review]
73. Pennesi M.E., Tan O., Parker M., Erker L., Bennett L.D., Huang D., Birch D.G., Heckenlively J.R., Chulay J.D., Wilson D.J. Investigative Ophthalmology and Visual Science. Conference: 2015 Annual Meeting of the Association for Research in Vision and Ophthalmology, ARVO 2015. United States. 56 (7) (pp 3835), 2015. Date of Publication: June 2015 Analysis of visual acuity and cyst volume measurements in a natural history study in X-Linked retinoschisis (XLRS).
74. Pennesi ME; Weleber RG; Yang P; Whitebirch C; Thean B; Flotte TR; Humphries M; Chegarnov E; Beasley KN; Stout JT; Chulay JD. Results at 5 Years After Gene Therapy for RPE65-Deficient Retinal Dystrophy.
75. Prokosch V., Stupp T., Spaniol K., Pham E., Nikol S. Journal of Gene Medicine. 16 (9–10) (pp 309–316), 2014. Date of Publication: 01 Sep 2014. Angiogenic gene therapy does not cause retinal pathology.
76. Rakoczy E., Lai M., Pierce C., Magno A., Samulski R., Chalberg T., Blumenkranz M., Constable I. Investigative Ophthalmology and Visual Science. Conference: 2013 Annual Meeting of the Association for Research in Vision and Ophthalmology, ARVO 2013. United States. 54 (15) (no pagination), 2013. Date of Publication: June 2013 Anti-VEGF Gene Therapy for Wet AMD: Phase I/II Safety and Pharmacology Results.
77. Rakoczy E., Magno A., Lai C.-M., Degli-Esposti M., Constable I. Investigative Ophthalmology and Visual Science. Conference: 2018 Annual Meeting of the Association for Research in Vision and Ophthalmology, ARVO 2018. United States. 59 (9) (no pagination), 2018. Date of Publication: July 2018 Subanalysis of data from rAAV.sFLT-1 phase 1 and 2a randomized gene therapy trials for wet age-related macular degeneration.
78. Rakoczy E.P., Lai C.-M., Magno A., French M., Butler S., Barone S., Schwartz S.D., Blumenkranz M., Degli-Esposti M., Constable I. Investigative Ophthalmology and Visual Science. Conference: 2017 Annual Meeting of the Association for Research in Vision and Ophthalmology, ARVO 2017. United States. 58 (8) (no pagination), 2017. Date of Publication: June 2017 Safety and post hoc analysis of subretinal rAAV.sFLT-1 for wet age-related macular degeneration following a phase 2a randomized clinical trial.
79. Rakoczy E.P., Lai C.-M., Magno A.L., Wikstrom M.E., French M.A., Pierce C.M., Schwartz S.D., Blumenkranz M.S., Chalberg T.W., Degli-Esposti M.A., Constable I.J. The Lancet. 386 (10011) (pp 2395–2403), 2015. Date of Publication: 01 Dec 2015 Gene therapy with recombinant adeno-associated vectors for neovascular age-related macular degeneration: 1 year follow-up of a phase 1 randomised clinical trial.
80. Rakoczy E.P., Lai M., Magno A.L., French M., Chalberg T.W., Blumenkranz M., Schwartz S.D., Constable I.J. Investigative Ophthalmology and Visual Science. Conference: 2014 Annual Meeting of the Association for Research in Vision and Ophthalmology, ARVO 2014. United States. 55 (13) (pp 1309), 2014. Date of Publication: April 2014. One year follow-up report on the rAAV.sFlt-1 phase i gene therapy trial for exudative age-related macular degeneration.
81. Rakoczy E.P., Magno A.L., Lai C.-M., Pierce C.M., Degli-Esposti M.A., Blumenkranz M.S., Constable I.J. American Journal of Ophthalmology. 204 (pp 113–123), 2019. Date of Publication: August 201 Three-Year Follow-Up of Phase 1 and 2a rAAV.sFLT-1 Subretinal Gene Therapy Trials for Exudative Age-Related Macular Degeneration.
82. Rasmussen H; Chu KW; Campochiaro P; Gehlbach PL; Haller JA; Handa JT; Nguyen QD; Sung JU. Human Gene Therapy. 12(16):2029–32, 2001 Nov 01 Clinical protocol. An open-label, phase I, single administration, dose-escalation study of ADGVPEDF.11D (ADPEDF) in neovascular age-related macular degeneration (AMD).
83. Ripamonti C; Henning GB; Robbie SJ; Sundaram V; van den Born LI; Casteels I; de Ravel TJ; Moore AT; Smith AJ; Bainbridge JW; Ali RR; Stockman A. Journal of Vision. 15(15):20, 2015 Spectral sensitivity measurements reveal partial success in restoring missing rod function with gene therapy.
84. Ripamonti C., Henning G.B., Robbie S.J., Sundaram V., van den Born L.I., Casteels I., de Ravel T.J., Moore A.T., Smith A.J., Bainbridge J.W., Ali R.R., Stockman A. Journal of vision. 15 (15) (pp 20), 2015. Date of Publication: 2015 Spectral sensitivity measurements reveal partial success in restoring missing rod function with gene therapy.
85. Russell S; Bennett J; Wellman JA; Chung DC; Yu ZF; Tillman A; Wittes J; Pappas J; Elci O; McCague S; Cross D; Marshall KA; Walshire J; Kehoe TL; Reichert H; Davis M; Raffini L; George LA; Hudson FP; Dingfield L; Zhu X; Haller JA; Sohn EH; Mahajan VB; Pfeifer W; Weckmann M; Johnson C; Gewaily D; Drack A; Stone E; Wachtel K; Simonelli F; Leroy BP; Wright JF; High KA; Maguire AM. Lancet. 390(10097):849–860, 2017 Aug 26. Efficacy and safety of voretigene neparvovec (AAV2-hRPE65v2) in patients with RPE65-mediated inherited retinal dystrophy: a randomised, controlled, open-label, phase 3 trial.
86. Russell S., Bennett J., Wellman J.A., Chung D.C., Yu Z.-F., Tillman A., Wittes J., Pappas J., Elci O., McCague S., Cross D., Marshall K.A., Walshire J., Kehoe T.L., Reichert H., Davis M., Raffini L., George L.A., Hudson F.P., Dingfield L., Zhu X., Haller J.A., Sohn E.H., Mahajan V.B., Pfeifer W., Weckmann M., Johnson C., Gewaily D., Drack A., Stone E., Wachtel K., Simonelli F., Leroy B.P., Wright J.F., High K.A., Maguire A.M. The Lancet. 390 (10097) (pp 849–860), 2017. Date of Publication: 26 August–1 September 2017 Efficacy and safety of voretigene neparvovec (AAV2-hRPE65v2) in patients with RPE65-mediated inherited retinal dystrophy: a randomised, controlled, open-label, phase 3 trial.
87. Russell S.R., Bennett J., Wellman J.A., Chung D.C., High K.A., Yu Z.-F., Tillman A., Maguire A.M. Investigative Ophthalmology and Visual Science. Conference: 2017 Annual Meeting of the Association for Research in Vision and Ophthalmology, ARVO 2017. United States. 58 (8) (no pagination), 2017. Date of Publication: June 2017 Year 2 results for a phase 3 trial of voretigene neparvovec in biallelic RPE65-mediated inherited retinal disease.
88. Salvetti A.P., Birtel J., Xue K., Gliem M., Mueller P., Holz F.G., MacLaren R.E., Issa P.C. Investigative Ophthalmology and Visual Science. Conference: 2017 Annual Meeting of the Association for Research in Vision and Ophthalmology, ARVO 2017. United States. 58 (8) (no pagination), 2017. Date of Publication: June 2017 Near-infrared autofluorescence in choroideremia: Anatomical and functional correlations.
89. Samuels B.C., Hammes N., Bernabe C., Federici L., Molosh A., Bhatnagar S., Shekhar A. Investigative Ophthalmology and Visual Science. Conference: 2017 Annual Meeting of the Association for Research in Vision and Ophthalmology, ARVO 2017. United States. 58 (8) (no pagination), 2017. Date of Publication: June 2017 Stimulation of hypothalamic orexin neurons using DREADD technology increases intraocular pressure.
90. Schuerch K., Lee W., Duncker T., Delori F.C., Allikmets R., Tsang S.H., Sparrow J.R. Investigative Ophthalmology and Visual Science. Conference: 2017 Annual Meeting of the Association for Research in Vision and Ophthalmology, ARVO 2017. United States. 58 (8) (no pagination), 2017. Date of Publication: June 2017. Longitudinal analysis of quantitative autofluorescence in recessive Stargardt disease (STGD1).
91. Seitz I.P., Jolly J.K., Dominik Fischer M., Simunovic M.P. Graefe’s Archive for Clinical and Experimental Ophthalmology. 256 (4) (pp 665–673), 2018. Date of Publication: 01 Apr 2018. Colour discrimination ellipses in choroideremia.
92. Simunovic MP; Jolly JK; Xue K; Edwards TL; Groppe M; Downes SM; MacLaren RE. Investigative Ophthalmology & Visual Science. 57(14):6033–6039, 2016 Nov 01 The Spectrum of CHM Gene Mutations in Choroideremia and Their Relationship to Clinical Phenotype.
93. Simunovic MP; Xue K; Jolly JK; JAMA Ophthalmology. 135(3):234–241, 2017 Mar 01. MacLaren RE. Structural and Functional Recovery Following Limited Iatrogenic Macular Detachment for Retinal Gene Therapy.
94. Sumaroka A; Cideciyan AV; Charng J; Wu V; Powers CA; Iyer BS; Lisi B; Swider M; Jacobson SG. Autosomal Dominant Retinitis Pigmentosa Due to Class B Rhodopsin Mutations: An Objective Outcome for Future Treatment Trials.
95. Tamai M. Nippon Ganka Gakkai zasshi. 108 (12) (pp 750–768; discussion 769), 2004. Date of Publication: Dec 2004 Progress in pathogenesis and therapeutic research in retinitis pigmentosa and age-related macular degeneration.
96. Testa F; Maguire AM; Rossi S; Pierce EA; Melillo P; Marshall K; Banfi S; Surace EM; Sun J; Acerra C; Wright JF; Wellman J; High KA; Auricchio A; Bennett J; Simonelli F. Ophthalmology. 120(6):1283–91, 2013 Jun Three-year follow-up after unilateral subretinal delivery of adeno-associated virus in patients with Leber congenital Amaurosis type 2.
97. Uretsky S., Vignal C., Thomasson N., Bouquet C., Galy A., Combal J.P., Fitoussi S., Sahel J.-A. Neurology. Conference: 69th American Academy of Neurology Annual Meeting, AAN 2017. United States. 88 (16 Supplement 1) (no pagination), 2017. Date of Publication: April 2017 Intravitreal rAAV2/2-ND4 (GS010): A gene therapy for vision loss in leber’s hereditary optic neuropathy (LHON) caused by the G11778A ND4 mitochondrial mutation.
98. Vignal C; Uretsky S; Fitoussi S; Galy A; Blouin L; Girmens JF; Bidot S; Thomasson N; Bouquet C; Valero S; Meunier S; Combal JP; Gilly B; Katz B; Sahel JA. Ophthalmology. 125(6):945–947, 2018 06 Safety of rAAV2/2-ND4 Gene Therapy for Leber Hereditary Optic Neuropathy.
99. Wan X; Pei H; Zhao MJ; Yang S; Hu WK; He H; Ma SQ; Zhang G; Dong XY; Chen C; Wang DW; Li B. Scientific Reports. 6:21587, 2016 Feb 19 Efficacy and Safety of rAAV2-ND4 Treatment for Leber’s Hereditary Optic Neuropathy.
100. Weleber R.G., Stout T., Lauer A.K., Pennesi M.E., Audo I.S., Mohand-Said S., Barale P.-O., Buggage R., Sahel J.A., Wilson D.J. Investigative Ophthalmology and Visual Science. Conference: 2015 Annual Meeting of the Association for Research in Vision and Ophthalmology, ARVO 2015. United States. 56 (7) (pp 2286), 2015. Date of Publication: June 2015 Early findings in a Phase I/IIa clinical program for Usher syndrome 1B (USH1B; MIM #276900).
101. Weleber RG; Pennesi ME; Wilson DJ; Kaushal S; Erker LR; Jensen L; McBride MT; Flotte TR; Humphries M; Calcedo R; Hauswirth WW; Chulay JD; Stout JT. Ophthalmology. 123(7):1606–20, 2016 07 Results at 2 Years after Gene Therapy for RPE65-Deficient Leber Congenital Amaurosis and Severe Early-Childhood-Onset Retinal Dystrophy.
102. Xue K; Jolly JK; Barnard AR; Rudenko A; Salvetti AP; Patricio MI; Edwards TL; Groppe M; Orlans HO; Tolmachova T; Black GC; Webster AR; Lotery AJ; Holder GE; Downes SM; Seabra MC; MacLaren RE. Nature Medicine. 24(10):1507–1512, 2018 10. Beneficial effects on vision in patients undergoing retinal gene therapy for choroideremia.
103. Xue K; Oldani M; Jolly JK; Edwards TL; Groppe M; Downes SM; MacLaren RE. Investigative Ophthalmology & Visual Science. 57(8):3674–84, 2016 07 01. Correlation of Optical Coherence Tomography and Autofluorescence in the Outer Retina and Choroid of Patients With Choroideremia.
104. Xue K., Jolly J.K., Barnard A.R., Rudenko A., Salvetti A.P., Patricio M.I., Edwards T.L., Groppe M., Orlans H.O., Tolmachova T., Black G.C., Webster A.R., Lotery A.J., Holder G.E., Downes S.M., Seabra M.C., MacLaren R.E. Nature Medicine. 24 (10) (pp 1507–1512), 2018. Date of Publication: 01 Oct 2018 Beneficial effects on vision in patients undergoing retinal gene therapy for choroideremia.
105. Xue K., Oldani M., Jolly J.K., Edwards T.L., Groppe M., Downes S.M., Maclaren R.E. Investigative Ophthalmology and Visual Science. 57 (8) (pp 3674–3684), 2016. Date of Publication: July 2016 Correlation of optical coherence tomography and autofluorescence in the outer retina and choroid of patients with choroideremia.
106. Yang S; Ma SQ; Wan X; He H; Pei H; Zhao MJ; Chen C; Wang DW; Dong XY; Yuan JJ; Li B. EBioMedicine. 10:258–68, 2016 Aug Long-term outcomes of gene therapy for the treatment of Leber’s hereditary optic neuropathy.
107. Yang S; Yang H; Ma SQ; Wang SS; He H; Zhao MJ; Li B. Medicine. 95(40):e5110, 2016 Oct Evaluation of Leber’s hereditary optic neuropathy patients prior to a gene therapy clinical trial.
108. Yang S., Yang H., Ma S.-Q., Wang S.-S., He H., Zhao M.-J., Li B. Medicine (United States). 95 (40) (no pagination), 2016. Article Number: e5110. Date of Publication: 2016 Evaluation of Leber’s hereditary optic neuropathy patients prior to a gene therapy clinical trial.
109. Yu-Wai-Man P. Neuro-Ophthalmology. Conference: 13th Meeting of the European Neuro-Ophthalmological Society, EUNOS 2017. Hungary. 41 (Supplement 1) (pp S41-S43), 2017. Date of Publication: September 2017 Preliminary baseline characteristics of patients with leber hereditary optic neuropathy (LHON) enrolled in the rescue and reverse phase III clinical gene therapy trials.
110. Yu-Wai-Man P., Moster M., Sadun A.A., Klopstock T., Vignal-Clermont C., Newman N.J., Sergott R.C., Carelli V., Chevalier C., Blouin L., Taiel M., Katz B., Sahel J.A. Investigative Ophthalmology and Visual Science. Conference: 2019 Annual Meeting Association for Research in Vision and Ophthalmology, ARVO 2019. Canada. 60 (9) (no pagination), 2019. Date of Publication: July 2019 RAAV2/2-ND4 for the Treatment of Leber Hereditary Optic Neuropathy (LHON): 72-Week Data from the REVERSE Phase III Clinical Trial.
111. Yu-Wai-Man P., Newman N.J., Sergott R., Bryan M.S., Carelli V., Klopstock T., Moster M., Sadun A.A., Sahel J.A., Uretsky S., Vignal C. Investigative Ophthalmology and Visual Science. Conference: 2017 Annual Meeting of the Association for Research in Vision and Ophthalmology, ARVO 2017. United States. 58 (8) (no pagination), 2017. Date of Publication: June 2017 Preliminary baseline characteristics of patients with leber hereditary optic neuropathy (LHON) enrolled in the RESCUE and REVERSE clinical gene therapy trials.
112. Yuan J; Zhang Y; Liu H; Tian Z; Li X; Zheng Y; Gao Q; Song L; Xiao X; Sun J; Wang Z; Li B. Current Gene Therapy. 18(6):386–392, 2018. Clinical Observation of Patients with Leber’s Hereditary Optic Neuropathy Before Gene Therapy.
113. Zinkernagel MS; Groppe M; MacLaren RE. Ophthalmology. 120(8):1592–6, 2013 Aug Macular hole surgery in patients with end-stage choroideremia.
114. Chung DC, Bertelsen M, Lorenz B, Pennesi ME, Leroy BP, Hamel CP, et al. The Natural History of Inherited Retinal Dystrophy Due to Biallelic Mutations in the RPE65 Gene. Am J Ophthalmol. 2019;
115. Yao-Yao Zhu. BLA Clinical Review Memorandum—No. 125610 (FDA) [Internet]. 2017. Available from: https://www.fda.gov/files/vaccines%2Cblood%26

### Appendix A.3. Selected Studies (6) for Meta-Analyses

(1) **Bainbridge et al., 2015 NEJM**. Long-Term Effect of Gene Therapy on Leber’s Congenital Amaurosis. James W B Bainbridge, Manjit S Mehat, Venki Sundaram, Scott J Robbie, Susie E Barker, Caterina Ripamonti, Anastasios Georgiadis, Freya M Mowat, Stuart G Beattie, Peter J Gardner, Kecia L Feathers, Vy A Luong, Suzanne Yzer, Kamaljit Balaggan, Ananth Viswanathan, Thomy J L de Ravel, Ingele Casteels, Graham E Holder, Nick Tyler, Fred W Fitzke, Richard G Weleber, Marko Nardini, Anthony T Moore, Debra A Thompson, Simon M Petersen-Jones, Michel Michaelides, L Ingeborgh van den Born, Andrew Stockman, Alexander J Smith, Gary Rubin, Robin R Ali. N Engl J Med 2015 May 14;372(20):1887–97, doi:10.1056/NEJMoa1414221. Epub 2015 May 4.5 [77].
(2) **Le Meur et al., 2018 Mol Ther**. Safety and Long-Term Efficacy of AAV4 Gene Therapy in Patients with RPE65 Leber Congenital Amaurosis. Guylène Le Meur, Pierre Lebranchu, Fanny Billaud, Oumeya Adjali, Sébastien Schmitt, Stéphane Bézieau, Yann Péréon, Romain Valabregue, Catherine Ivan, Christophe Darmon, Philippe Moullier, Fabienne Rolling, Michel Weber. Mol Ther. 2018 Jan 3;26(1):256–268, doi:10.1016/j.ymthe.2017.09.014. Epub 2017 Sep 19 [78].
(3) **Jacobson et al., 2012 Arch Ophthalmol**. Gene Therapy for Leber Congenital Amaurosis Caused by RPE65 Mutations-Safety and Efficacy in 15 Children and Adults Followed Up to 3 Years. Samuel G Jacobson, Artur V Cideciyan, Ramakrishna Ratnakaram, Elise Heon, Sharon B Schwartz, Alejandro J Roman, Marc C Peden, Tomas S Aleman, Sanford L Boye, Alexander Sumaroka, Thomas J Conlon, Roberto Calcedo, Ji-Jing Pang, Kirsten E Erger, Melani B Olivares, Cristina L Mullins, Malgorzata Swider, Shalesh Kaushal, William J Feuer, Alessandro Iannaccone, Gerald A Fishman, Edwin M Stone, Barry J Byrne, William W Hauswirth; Arch Ophthalmol. 2012 Jan;130(1):9–24, doi:10.1001/archophthalmol.2011.298. Epub 2011 Sep 12. [79].
(4) **Russell et al., 2017 The Lancet**. Efficacy and safety of voretigene neparvovec (AAV2-hRPE65v2) in patients with RPE65-mediated inherited retinal dystrophy: a randomised, controlled, open-label, phase 3 trial. Stephen Russell, Jean Bennett, Jennifer A Wellman, Daniel C Chung, Zi-Fan Yu, Amy Tillman, Janet Wittes, Julie Pappas, Okan Elci, Sarah McCague, Dominique Cross, Kathleen A Marshall, Jean Walshire, Taylor L Kehoe, Hannah Reichert, Maria Davis, Leslie Raffini, Lindsey A George, F Parker Hudson, Laura Dingfield, Xiaosong Zhu, Julia A Haller, Elliott H Sohn, Vinit B Mahajan, Wanda Pfeifer, Michelle Weckmann, Chris Johnson, Dina Gewaily, Arlene Drack, Edwin Stone, Katie Wachtel, Francesca Simonelli, Bart P Leroy, J Fraser Wright, Katherine A High, Albert M Maguire. 2017 Aug 26;390(10097):849–860, doi:10.1016/S0140-6736(17)31868-8. Epub 2017 Jul 14. [58].
(5) **Testa et al., 2013 Ophthalmology.** Three-Year Follow-up after Unilateral Subretinal Delivery of Adeno-Associated Virus in Patients with Leber Congenital Amaurosis Type 2. Francesco Testa, Albert M Maguire, Settimio Rossi, Eric A Pierce, Paolo Melillo, Kathleen Marshall, Sandro Banfi, Enrico M Surace, Junwei Sun, Carmela Acerra, J Fraser Wright, Jennifer Wellman, Katherine A High, Alberto Auricchio, Jean Bennett, Francesca Simonelli. Ophthalmology, 2013 Jun;120(6):1283–91, doi:10.1016/j.ophtha.2012.11.048. Epub 2013 Mar 6. [80].
(6) **Weleber et al., 2016 Ophthalmology.** Results at 2 Years after Gene Therapy for RPE65-Deficient Leber Congenital Amaurosis and Severe Early-Childhood Onset Retinal Dystrophy. Richard G Weleber, Mark E Pennesi, David J Wilson, Shalesh Kaushal, Laura R Erker, Lauren Jensen, Maureen T McBride, Terence R Flotte, Margaret Humphries, Roberto Calcedo, William W Hauswirth, Jeffrey D Chulay, J Timothy Stout. Ophthalmology 2016 Jul;123(7):1606–20, doi:10.1016/j.ophtha.2016.03.003. Epub 2016 Apr 19. [81].

### Appendix A.4. Summary Trial Inclusion, Exclusion Eligibility and Endpoints

**Table A1:** Summary Trial Inclusion, Exclusion Eligibility and Endpoints.

Study Author		Bainbridge et al., 2015 (NEJM). NCT00643747		Jacobson et al., 2012 (Arch Ophthalmol). NCT00481546		Le Meur et al., 2018 (Mol Ther). NCT01496040		Russell et al., 2017 (Lancet). NCT00999609		Testa et al., 2013 (Ophthalmology). NCT00516477		Weleber et al., 2016 (Ophthalmology). NCT00749957
**Principal Investigator**		Study Director: **Robin R Ali**, PhD University College, London		**Samuel G. Jacobson**, MD, PhD; University of Pennsylvania		**Michel WEBER**, Professor; CHU Nantes		**Albert M Maguire**, MD (Children’s Hospital of Philadelphia) and **Stephen R Russell**, MD (University of Iowa).		Study Director: Clinical Director, Spark Therapeutics		**J Timothy Stout**, MD, PhD, MBA; Casey Eye Institute, Oregon Health & Science University
**Sponsor (Academic/Industry)**		University College, London; (Moorfields Eye Hospital NHS Foundation Trust; Targeted Genetics Corporation)		University of Pennsylvania		Nantes University Hospital		Spark Therapeutics		Spark Therapeutics		Applied Genetic Technologies Corporation; (Oregon Health and Science University; University of Massachusetts, Worcester.
**Official title**		An Open-Label Dose Escalation Study of an Adeno-associated Virus Vector (AAV2/2-hRPE65p-hRPE65) for Gene Therapy of Severe Early-Onset Retinal Degeneration		Phase I Trial of Ocular Subretinal Injection of a Recombinant Adeno-Associated Virus (rAAV2-CBSB-hRPE65) Gene Vector to Patients With Retinal Disease Due to RPE65 Mutations (Clinical Trials of Gene Therapy for Leber Congenital Amaurosis)		Prospective Monocentric Open Label Non Randomized Uncontrolled Phase I/II Clinical Gene Therapy Protocol for the Treatment of Retinal Dystrophy Caused by Defects in RPE65		A Safety and Efficacy Study in Subjects With Leber Congenital Amaurosis (LCA) Using Adeno-Associated Viral Vector to Deliver the Gene for Human RPE65 to the Retinal Pigment Epithelium (RPE) [AAV2-hRPE65v2-301]		A Phase 1 Safety Study in Subjects With Leber Congenital Amaurosis (LCA) Using Adeno-Associated Viral Vector to Deliver the Gene for Human RPE65 Into the Retinal Pigment Epithelium (RPE) [AAV2-hRPE65v2-101]		A Multiple-Site, Phase 1/2, Safety and Efficacy Trial of a Recombinant Adeno-associated Virus Vector Expressing RPE65 (rAAV2-CB-hRPE65) in Patients With Leber Congenital Amaurosis Type 2
**Study design**		Phase 1–2, open-label, non-randomized;		Phase 1, open-label, non-randomized;		Phase 1/2, open, non-randomized;		Phase 3, open-labelled, randomised (RCT);		Phase 1, open-label, non-randomized (3-year study);		Phase 1–2, open-label, non-randomized;
**Treatment**		rAAV 2/2. hRPE65p.hRPE65		rAAV2-RPE65		AAV2/4.-RPE65-RPE65		AAV2-hRPE65v2		AAV2-hRPE65v2		rAAV2-CB-hRPE65
**Inclusion Criteria:**	**1**	Clinical diagnosis of severe early-onset retinal dystrophy confirmed missense mutation(s) in RPE65	**1**	RPE65-associated retinal disease (two disease-causing RPE65 mutations);	**1**	Mutations that code for abnormal RPE65 protein	**1**	Willingness to adhere to protocol and long-term follow-up as evidenced by written informed consent or parental permission and subject assent (where applicable).	**1**	Male and female subjects of any ethnic group are eligible for participation in this study, providing they meet the following criteria:	**1**	Retinal disease consistent with a diagnosis of Leber congenital amaurosis and documented mutations in the RPE65 gene (including null mutations and mutations that code for abnormal RPE65 protein);
			**2**	Clinical diagnosis of Leber congenital amaurosis (LCA)/early-onset retinal degeneration (EORD) and of severely impaired visual and retinal function, and best corrected visual acuity of 20/40 or worse in the study eye;	**2**	Presence of characteristic abnormalities in fundus	**2**	Diagnosis of LCA due to RPE65 mutations; molecular diagnosis is to be performed, or confirmed, by a CLIA-approved laboratory.	**2**	Must be willing to adhere to protocol and companion protocol for long-term follow-up as evidenced by written informed consent or parental permission and subject assent.	**2**	At least 6 years of age;
			**3**	Ability to perform tests of visual and retinal function;	**3**	Dramatic reduction of both rods ans cones ERG responses	**3**	Age three years old or older.	**3**	Adults and children diagnosed with LCA.	**3**	Good general health without significant physical examination findings or clinically significant abnormal laboratory results;
			**4**	Visible photoreceptor layer on a standard OCT scan;	**4**	Low visual acuity <0.32	**4**	Visual acuity worse than 20/60 (both eyes) and/or visual field less than 20 degrees in any meridian as measured by a III4e isopter or equivalent (both eyes).	**4**	Molecular diagnosis of LCA due to RPE65 mutations (homozygotes or compound heterozygotes) by a CLIA-approved laboratory.	**4**	Able to perform tests of visual and retinal function;
			**5**	Good general health;	**5**	inform consent signed	**5**	Sufficient viable retinal cells as determined by non-invasive means, such as optical coherence tomography (OCT) and/or ophthalmoscopy. Must have either: 1) an area of retina within the posterior pole of >100 µm thickness shown on OCT; 2) ≥ 3 disc areas of retina without atrophy or pigmentary degeneration within the posterior pole; or 3) remaining visual field within 30 degrees of fixation as measured by a III4e isopter or equivalent.	**5**	Age eight years old or older at the time of administration.	**5**	Visual acuity not better than 20/60 and not worse than hand motion in both the treated eye and the fellow eye;
			**6**	Ability to comply with research procedures;			**6**	Subjects must be evaluable on mobility testing (the primary efficacy endpoint) to be eligible for the study. Evaluable is defined as: 1) The ability to perform mobility testing within the luminance range evaluated in the study. Individuals must receive an accuracy score of ≤ 1 during screening mobility testing at 400 lux or less to be eligible; individuals with an accuracy score of > 1 on all screening mobility test runs at 400 lux, or those who refuse to perform mobility testing at screening, will be excluded. 2) The inability to pass mobility testing at 1 lux. Individuals must fail screening mobility testing at 1 lux to be eligible; individuals that pass one or more screening mobility test runs at 1 lux will be excluded.	**6**	Visual acuity ≤ 20/160 or visual field less than 20 degrees in the eye to be injected.	**6**	Visible photoreceptor (outer nuclear) layer on a standard optical coherence tomography (OCT) scan;
			**7**	Specific for Cohorts 1, 2 and 4: 18 years of age and older;							**7**	Acceptable hematology, clinical chemistry and urine laboratory parameters;
			**8**	Specific for Cohorts 3 and 5: Between 8 and 17 years of age, inclusive.							**8**	For females of childbearing potential, a negative pregnancy test at screening and at baseline, and agreement to use effective contraception for 12 months after administration of rAAV2-CB-hRPE65, for sexual activity that could lead to pregnancy;
											**9**	For males of reproductive potential, agreement to use effective contraception for 12 months after administration of rAAV2-CB-hRPE65, for sexual activity that could lead to pregnancy
**Exclusion Criteria:**	**1**	Visual acuity in the study eye better than 6/36 Snellen	**1**	AAV antibody titers greater than two standard deviations above normal at baseline;	**1**	Patients with chronic conditions such a haematological, cardiac, renal diseases	**1**	Unable or unwilling to meet requirements of the study, including receiving bilateral subretinal vector administrations.	**1**	SUBJECTS WILL NOT BE EXCLUDED BASED ON THEIR GENDER, RACE OR ETHNICITY. Subjects who meet any of the following conditions are excluded from the clinical study: Subjects who meet any of the following conditions are excluded from the clinical study:	**1**	Pre-existing eye conditions that would preclude the planned surgery or interfere with interpretation of study endpoints or complications of surgery (e.g., glaucoma, corneal or lenticular opacities, or history or retinal detachment);
	**2**	Hypertension	**2**	Humoral immune deficiency as evidenced by low tetanus toxoid IgG antibody titers;	**2**	Patients with, within the past 6 months, a clinically significant cardiac disease or known congestive heart failure, cardiac rhytm and conduction abnormalities	**2**	Any prior participation in a study in which a gene therapy vector was administered.	**2**	Unable or unwilling to meet requirements of the study.	**2**	Presence of epiretinal membrane on OCT;
	**3**	Diabetes mellitus	**3**	Pre-existing eye conditions that would preclude the planned surgery or interfere with the interpretation of study endpoints or surgical complications;	**3**	Patients with pulmonaty dysfunction	**3**	Participation in a clinical study with an investigational drug in the past six months.	**3**		**3**	History of immunodeficiency or other medical conditions that might increase the risk of rAAV2-CB-hRPE65 administration;
	**4**	Tuberculosis	**4**	Complicating systemic diseases;	**4**	Patients with suspected rheumatoid arthritis	**4**	Use of retinoid compounds or precursors that could potentially interact with the biochemical activity of the RPE65 enzyme; individuals who discontinue use of these compounds for 18 months may become eligible.	**4**	Participation in a clinical study with an investigational drug in the past six months.	**4**	Use of anticoagulants or anti-platelet agents within 7 days prior to study agent administration;
	**5**	Renal impairment	**5**	Use of anti-platelet agents that may alter coagulation within 7 days prior to study agent administration;	**5**	Patients with current systemic infection……..	**5**	Prior intraocular surgery within six months.	**5**	Pre-existing eye conditions that would preclude the planned surgery or interfere with the interpretation of study endpoints (for example, glaucoma, corneal or lenticular opacities).	**5**	History of allergy or sensitivity to medications planned for use in the peri-operative period;
	**6**	Immunocompromise	**6**	Use of immunosuppressive medications;			**6**	Known sensitivity to medications planned for use in the peri-operative period.	**6**	Lack of sufficient viable retinal cells as determined by non-invasive means, such as optical coherence tomography (OCT) and/or ophthalmoscopy. Specifically, if indirect ophthalmoscopy reveals less than 1 disc area of retina which is not involved by complete retinal degeneration (indicated by geographic atrophy, thinning with tapetal sheen, or confluent intraretinal pigment migration), these eyes will be excluded. In addition, in eyes where optical coherence tomography (OCT) scans of sufficient quality can be obtained, areas of retina with thickness measurements less than 100 um, or absence of neural retina, will not be targeted for delivery of AAV2-hRPE65v2.	**6**	For females of childbearing potential, a positive pregnancy test at screening or baseline (within 2 days before rAAV2-CB-hRPE65 administration);
	**7**	Osteoporosis	**7**	Pregnancy or breastfeeding;			**7**	Pre-existing eye conditions or complicating systemic diseases that would preclude the planned surgery or interfere with the interpretation of study. Complicating systemic diseases would include those in which the disease itself, or the treatment for the disease, can alter ocular function. Examples are malignancies whose treatment could affect central nervous system function (for example: radiation treatment of the orbit; leukemia with CNS/optic nerve involvement). Subjects with diabetes or sickle cell disease would be excluded if they had any manifestation of advanced retinopathy (e.g., macular edema or proliferative changes). Also excluded would be subjects with immunodeficiency (acquired or congenital) as there could be susceptibility to opportunistic infection (such as CMV retinitis).	**7**	Complicating systemic diseases or clinically significant abnormal baseline laboratory values. Complicating systemic diseases would include those in which the disease itself, or the treatment for the disease, can alter ocular function. Examples are malignancies whose treatment could affect central nervous system function (for example, radiation treatment of the orbit; leukemia with CNS/optic nerve involvement). Also excluded would be subjects with immuno-compromising diseases, as there could be susceptibility to opportunistic infection (such as CMV retinitis). Subjects with diabetes or sickle cell disease would be excluded if they had any manifestation of advanced retinopathy (e.g., macular edema or proliferative changes). Subjects with juvenile rheumatoid arthritis could be excluded due to increased infection risk after surgery due to poor wound healing. Subjects who are positive for hepatitis B, C, and HIV will be excluded.	**7**	Females who are breast feeding;
	**8**	Gastric ulceration	**8**	Individuals (males and females) of childbearing potential who are unwilling to use effective contraception;			**8**	Individuals of childbearing potential who are pregnant or unwilling to use effective contraception for four months following vector administration.	**8**	Prior ocular surgery within six months.	**8**	Use of any investigational agent, or systemic corticosteroids or other immunosuppressive drug(s), within 3 months prior to enrollment;
	**9**	Severe affective disorder)	**9**	Any condition that would prevent a subject from completing follow-up examinations during the course of the study;			**9**	Individuals incapable of performing mobility testing (the primary efficacy endpoint) for reason other than poor vision, including physical or attentional limitations.	**9**	Known sensitivity to medications planned for use in the peri-operative period.	**9**	Prior receipt of any AAV gene therapy product;
	**10**	Pregnancy or lactation	**10**	Any condition that makes the subject unsuitable for the study;			**10**	Any other condition that would not allow the potential subject to complete follow-up examinations during the course of the study or, in the opinion of the investigator, makes the potential subject unsuitable for the study.	**10**	Individuals of childbearing potential who are pregnant or unwilling to use effective contraception for the duration of the study.	**10**	Any condition which leads the investigator to believe that the participant cannot comply with the protocol requirements or that may place the participant at an unacceptable risk for participation.
			**11**	Current, or recent participation, in any other research protocol involving investigational agents or therapies;			**11**	Subjects will not be excluded based on their gender, race, or ethnicity.	**11**	Any other condition that would not allow the potential subject to complete follow-up examinations during the course of the study and, in the opinion of the investigator, makes the potential subject unsuitable for the study.		
			**12**	Recent receipt of an investigational biologic therapeutic agent.					**12**	Subjects will be excluded if immunological studies show presence of neutralizing antibodies to AAV2 above 1:1000.		
**Primary Outcome Measures:**	**1**	intraocular inflammation [Time Frame: at intervals up to 12 months]	**1**	The primary safety endpoint in this trial is the standard ocular examination. Toxicity will also be assessed by measurement of vision, hematology and serum chemistries, assays for vector genomes, reported subject history of symptoms and adverse events. [Time Frame: 15 years]	**1**	The drug safety evaluation after administration [Time Frame: After administration of the gene therapy product.The patient will be folloed for the duration of the hospital stay, an average of 7 days]	**1**	Multi-luminance Mobility Testing (MLMT), Bilateral [Time Frame: One year (change from baseline)]; The MLMT measures changes in functional vision, as assessed by the ability to navigate a course accurately and at a reasonable pace at different levels of environmental illumination. MLMT was assessed **using both eyes** at 1 or more of 7 levels of illumination, ranging from 400 lux (a brightly lit office) to 1 lux (a moonless summer night). Each light level was assigned a score code ranging from 0 to 6. A higher score indicated that a subject was able to pass the MLMT at a lower light level. A score of −1 was assigned to those who could not pass MLMT at 400 lux. The MLMT of each subject was videotaped and assessed by independent graders. The MLMT score was determined by the lowest light level at which the subject was able to pass the MLMT. The MLMT score change was defined as the difference between the score at Baseline and the score at Year 1. A positive MLMT score change from Baseline to Year 1 visit indicated that the subject was able to complete the MLMT at a lower light level.	**1**	The primary outcome measures are safety and tolerability. Secondary outcome measure(s) include changes in visual function as measured by subjective, psychophysical tests and by objective, physiologic tests. [Time Frame: Visual function will be measured at designated intervals from baseline visits through 5 years as stated in the protocol.]	**1**	Number of Participants Experiencing Ocular or Non-ocular Adverse Events [Time Frame: 2 years]
**Secondary Outcome Measures:**	**1**	visual function [Time Frame: intervals up to 12 months]	**1**	Visual function will be quantified prior to and after vector administration in order to determine whether vector administration affects visual function. [Time Frame: 15 years]	**2**	Biodistribution: Urine sampling and nasal secretion will be collected at several time points after administration of the gene therapy product during all the duration of hospital stay, an average of 7 days.	**1**	Full-field Light Sensitivity Threshold (FST) Testing: White Light [Time Frame: One year (change from baseline)]; Measures the light sensitivity of the entire visual field by recording the luminance at which a subject reliably reports seeing the dimmest flash.			**2**	Participants With Changes in Visual Fields [Time Frame: 2 years]; Improvement in the central 30 degree visual field, measured by static perimetry, at one or more time points after treatment, that was greater than the limit of agreement for baseline values.
					**3**	Different efficacy parameters and immune parameters have to be measured to conclude on the overall amelioration of quality of life of enrolled patients [Time Frame: Between Day −120 and Day −7, Day 5, Day 14, Day 30 Day 60, Day 90, Day 120, Day 180, Day 360]	**2**	Multi-luminance Mobility Testing (Monocular) [Time Frame: One year (change from baseline)]; The MLMT measures changes in functional vision, as assessed by the ability to navigate a course accurately and at a reasonable pace at different levels of environmental illumination. MLMT was assessed **using the first eye** at 1 or more of 7 levels of illumination, ranging from 400 lux (a brightly lit office) to 1 lux (a moonless summer night). Each light level was assigned a score code ranging from 0 to 6. A higher score indicated that a subject was able to pass the MLMT at a lower light level. A score of -1 was assigned to those who could not pass MLMT at 400 lux. The MLMT of each subject was videotaped and assessed by independent graders. The MLMT score was determined by the lowest light level at which the subject was able to pass the MLMT. The MLMT score change was defined as the difference between the score at Baseline and the score at Year 1. A positive MLMT score change from Baseline to Year 1 visit indicated that the subject was able to complete the MLMT at a lower light level.			**3**	Participants With Changes in Best Corrected Visual Acuity [Time Frame: 2 years]; Increase in BCVA of 7 or more letters at Year 2 visit compared to average baseline value
					**4**	Recording global ERG (electroretinogram)	**3**	Visual Acuity [Time Frame: One year (change from baseline)]; Measurement of the sharpness of vision, determined by the ability to read letters on a standardized chart from a specified distance.				
					**5**	Patient efficacy questionnaire						
					**6**	Testing of far and near visual acuity, color vision, pupillometry, microperimetry and dark adaptation.						

### Appendix A.5. ROBINS-I Risk of Bias in Non-Randomised Studies of Interventions

**Table A2:** ROBINS-I Risk of Bias in Non-Randomised Studies of Interventions.

	ROBINS-I Risk of Bias in Non-Randomised Studies of Interventions (NRSI)										
(1) Yes[Y]; (2) Probably Yes[PY]; (3) Probably no[PN]; (4) No[No]; and (5) No information[NI].											
Risk of bias assessment		A		B		C		D		E	
Responses underlined in green are potential markers for low risk of bias, and responses in red are potential markers for a risk of bias. Where questions relate only to sign posts to other questions, no formatting is used.		Bainbridge et al., 2015		Jacobson et al., 2012		Le Meur et al. , 2018		Testa et al. , 2013		Weleber et al., 2016	
	**Signalling questions**	**Description**	**Response options**	**Description**	**Response options**	**Description**	**Response options**	**Description**	**Response options**	**Description**	**Response options**
**Bias due to confounding**											
	1.1 Is there potential for confounding of the effect of intervention in this study?	**4** of the **12** patients used the better eye, contradicting the text suggesting that the poorer eye should be the treated eye, Table 1 (page 1889); the conentration of vector genomes (*vg*) have different cohorts;	**Y**	Y; “worse” vs. “better” eyes was logMAR 1.09 vs. 0.96, respectively, and there was no adjustment;	**Y**	Y; “worse” had an avarage of 31.5 letters while “better”eyes had 41.1 letters and there was no adjustment;	**Y**	Y; “worse” had a logMAR of 1.47 while “better”eyes had 1.14 logMAR, and there was no adjustment;	**Y**	Y; “worse” had an avarage of 24.0 letters while “better”eyes had 28.4 letters and there was no adjustment;	**Y**
	1.2. Was the analysis based on splitting participants’ follow up time according to intervention received?	Not significant time split between control vs. intervention;	**N**		**N**		**N**		**N**		**N**
	1.3. Were intervention discontinuations or switches likely to be related to factors that are prognostic for the outcome?		**NI**		**NI**		**NI**		**NI**		**NI**
	**Questions relating to baseline confounding only**										
	1.4. Did the authors use an appropriate analysis method that controlled for all the important confounding domains?	There was no information that controlled a confounding domain;	**N**	See comment on Jacobson, page 21;	**N**		**N**		**N**		**N**
	1.5. **If Y/PY to 1.4**: Were confounding domains that were controlled for measured validly and reliably by the variables available in this study?	There was no contolled confounding domains.	**N**		**N**		**N**		**N**		**N**
	1.6. Did the authors control for any post-intervention variables that could have been affected by the intervention?	No information;	**NI**		**N**		**NI**		**NI**		**NI**
	**Questions relating to baseline and time-varying confounding**										
	1.7. Did the authors use an appropriate analysis method that controlled for all the important confounding domains and for time-varying confounding?	There is no control or adjustment that might impact the outcome between the worse eye vs. the better eye;	**N**		**N**		**N**		**N**		**N**
	1.8. **If Y/PY to 1.7**: Were confounding domains that were controlled for measured validly and reliably by the variables available in this study?	N, see comment above re: better eye of 4 patients were used;	**N**		**N**		**N**		**N**		**N**
	**Risk of bias judgement**	Moderate/(?); confounding occurs due to uncorrected baselines for treated vs. control;	**Low/Moderate**		**Low/Moderate**		**Low/Moderate**		**Low/Moderate**		**Low/Moderate**
	Optional: What is the predicted direction of bias due to confounding?	Unpredictable/Favours experimental	**Unpredictable**		**Unpredictable**		**Unpredictable**		**Unpredictable**		**Unpredictable**
**Bias in selection of participants into the study**		**Bainbridge ** **et al., 2015**		**Jacobson ** **et al., 2012**		**Le Meur ** **et al., 2018**		**Testa ** **et al., 2013**		**Weleber ** **et al., 2016**	
	2.1. Was selection of participants into the study (or into the analysis) based on participant characteristics observed after the start of intervention?		**N**		**N**		**N**		**N**		**N**
	2.2. **If Y/PY to 2.1**: Were the post-intervention variables that influenced selection likely to be associated with intervention?		**N**		**N**		**N**		**N**		**N**
	2.3 **If Y/PY to 2.2**: Were the post-intervention variables that influenced selection likely to be influenced by the outcome or a cause of the outcome?		**N**		**N**		**N**		**N**		**N**
	2.4. Do start of follow-up and start of intervention coincide for most participants?		**NI**		**NI**		**NI**		**NI**		**NI**
	2.5. **If Y/PY to 2.2 and 2.3, or N/PN to 2.4**: Were adjustment techniques used that are likely to correct for the presence of selection biases?		**NI**		**N**		**NI**		**NI**		**NI**
	**Risk of bias judgement**	Low	**Low**		**Low**		**Low**		**Low**		**Low**
	Optional: What is the predicted direction of bias due to selection of participants into the study?		**Towards null**		**Towards null**		**Towards null**		**Towards null**		**Towards null**
**Bias in classification of interventions**		**Bainbridge ** **et al., 2015**		**Jacobson ** **et al., 2012**		**Le Meur ** **et al., 2018**		**Testa ** **et al., 2013**		**Weleber ** **et al., 2016**	
	3.1 Were intervention groups clearly defined?		**Y**		**Y**		**Y**		**Y**		**Y**
	3.2 Was the information used to define intervention groups recorded at the start of the intervention?		**Y**		**Y**		**Y**		**Y**		**Y**
	3.3 Could classification of intervention status have been affected by knowledge of the outcome or risk of the outcome?		**PN**		**PN**		**PN**		**PN**		**PN**
	**Risk of bias judgement**	Low/Moderate	**Low**		**Low**		**Low**		**Low**		**Low**
	Optional: What is the predicted direction of bias due to classification of interventions?		**Towards null**		**Towards null**		**Towards null**		**Towards null**		**Towards null**
**Bias due to deviations from intended interventions**		**Bainbridge ** **et al., 2015**		**Jacobson ** **et al., 2012**		**Le Meur ** **et al., 2018**		**Testa ** **et al., 2013**		**Weleber ** **et al., 2016**	
	**If your aim for this study is to assess the effect of assignment to intervention, answer questions 4.1 and 4.2**										
	4.1. Were there deviations from the intended intervention beyond what would be expected in usual practice?		**N**		**N**		**N**		**N**		**N**
	4.2. **If Y/PY to 4.1**: Were these deviations from intended intervention unbalanced between groups *and* likely to have affected the outcome?		**N**		**N**		**N**		**N**		**N**
	**If your aim for this study is to assess the effect of starting and adhering to intervention, answer questions 4.3 to 4.6**										
	4.3. Were important co-interventions balanced across intervention groups?		**NI**		**NI**		**NI**		**NI**		**NI**
	4.4. Was the intervention implemented successfully for most participants?		**PY**		**PY**		**PY**		**PY**		**PY**
	4.5. Did study participants adhere to the assigned intervention regimen?		**Y**		**Y**		**Y**		**Y**		**Y**
	4.6. **If N/PN to 4.3, 4.4 or 4.5**: Was an appropriate analysis used to estimate the effect of starting and adhering to the intervention?		**Y**		**Y**		**Y**		**Y**		**Y**
	**Risk of bias judgement**	Low	**Low**		**Low**		**Low**		**Low**		**Low**
	Optional: What is the predicted direction of bias due to deviations from the intended interventions?		**Towards null**		**Towards null**		**Towards null**		**Towards null**		**Towards null**
**Bias due to missing data**		**Bainbridge ** **et al., 2015**		**Jacobson ** **et al., 2012**		**Le Meur ** **et al., 2018**		**Testa ** **et al., 2013**		**Weleber ** **et al., 2016**	
	5.1 Were outcome data available for all, or nearly all, participants?	N/PN; some partial missing data (see mobility data in Figure 2, pg 1892);	**Y**		**Y**		**Y**		**Y**		**Y**
	5.2 Were participants excluded due to missing data on intervention status?										
			**N**		**N**		**N**		**N**		**N**
	5.3 Were participants excluded due to missing data on other variables needed for the analysis?										
			**NI**		**NI**		**NI**		**NI**		**NI**
	5.4 **If PN/N to 5.1, or Y/PY to 5.2 or 5.3**: Are the proportion of participants and reasons for missing data similar across interventions?		**NA**		**NA**		**NA**		**NA**		**NA**
	5.5 **If PN/N to 5.1, or Y/PY to 5.2 or 5.3**: Is there evidence that results were robust to the presence of missing data?		**NI**		**NI**		**NI**		**NI**		**NI**
	**Risk of bias judgement**	Low	**Low**		**Low**		**Low**		**Low**		**Low**
	Optional: What is the predicted direction of bias due to missing data?		**Towards null**		**Towards null**		**Towards null**		**Towards null**		**Towards null**
**Bias in measurement of outcomes**		**Bainbridge ** **et al., 2015**		**Jacobson ** **et al., 2012**		**Le Meur ** **et al., 2018**		**Testa ** **et al., 2013**		**Weleber ** **et al., 2016**	
	6.1 Could the outcome measure have been influenced by knowledge of the intervention received?		**NI**		**NI**		**NI**		**NI**		**NI**
	6.2 Were outcome assessors aware of the intervention received by study participants?		**NI**		**NI**		**NI**		**NI**		**NI**
	6.3 Were the methods of outcome assessment comparable across intervention groups?		**Y**		**Y**		**Y**		**Y**		**Y**
	6.4 Were any systematic errors in measurement of the outcome related to intervention received?		**PN**		**PN**		**PN**		**PN**		**PN**
	**Risk of bias judgement**		**Low**		**Low**		**Low**		**Low**		**Low**
	Optional: What is the predicted direction of bias due to measurement of outcomes?		**Towards null**		**Towards null**		**Towards null**		**Towards null**		**Towards null**
**Bias in selection of the reported result**		**Bainbridge ** **et al., 2015**		**Jacobson ** **et al., 2012**		**Le Meur ** **et al., 2018**		**Testa ** **et al., 2013**		**Weleber ** **et al., 2016**	
	Is the reported effect estimate likely to be selected, on the basis of the results, from...										
	7.1 … multiple outcome *measurements* within the outcome domain?	Y; some outcomes were reported to collect some data (e.g., ERG, contrast sensitivity, or FAF) but the outcomes did not show the data; see this main paper of Tuohy & Megaw, Figure 2, showing 23 assays, arranged alphabetically; estimate of 10 of 23 outcomes identified;	**NI**	See this main paper of Tuohy & Megaw, Figure 2, showing 23 assays, arranged alphabetically; estimate of 8 of 23 outcomes identified;	**NI**	See this main paper of Tuohy & Megaw, Figure 2, showing 23 assays, arranged alphabetically; estimate of 6 of 23 outcomes identified;	**NI**	See this main paper of Tuohy & Megaw, Figure 2, showing 23 assays, arranged alphabetically; estimate of 9 of 23 outcomes identified;	**NI**	See this main paper of Tuohy & Megaw, Figure 2, showing 23 assays, arranged alphabetically; estimate of 5 of 23 outcomes identified;	**NI**
	7.2 … multiple *analyses* of the intervention-outcome relationship?	Y; there was no adjusted data	**NI**		**NI**		**NI**		**NI**		**NI**
	7.3 … different *subgroups*?		**NI**		**NI**		**NI**		**NI**		**NI**
	**Risk of bias judgement**	Low/Moderate	**Low**		**Low**		**Low**		**Low**		**Low**
	Optional: What is the predicted direction of bias due to selection of the reported result?	Towards null/Unpredictable	**Towards null**		**Towards null**		**Towards null**		**Towards null**		**Towards null**
**Overall bias**		**Bainbridge ** **et al., 2015**		**Jacobson ** **et al., 2012**		**Le Meur ** **et al., 2018**		**Testa ** **et al., 2013**		**Weleber ** **et al., 2016**	
	**Risk of bias judgement**		**Low**		**Low**		**Low**		**Low**		**Low**
	Optional: What is the overall predicted direction of bias for this outcome?		**Towards null**		**Towards null**		**Towards null**		**Towards null**		**Towards null**

### Appendix A.6. RoB-2—Risk of Bias in Randomised Studies of Interventions

**Table A3:** RoB-2—Risk of Bias in Randomised Studies of Interventions.

Cochrane Tool for RoB-2—Risk of Bias in Randomised Studies of Interventions (RoB 2)			
(1) Yes[Y]; (2) Probably Yes[PY]; (3) Probably No[PN]; (4) No[No]; and (5) No Information[NI].			
**Domain 1: Risk of bias arising from the randomization process**			
**Signalling questions**	**Comments**	**Response options**	**Actual responses—Russell et al., 2017**
1.1 Was the allocation sequence random?	The study was randomized; the allocation sequence was performed under direction of an independent biostatistician assigned to either intervention or control and the list of patients was created before enrolment.	Y/PY/PN/N/NI	**Y**
1.2 Was the allocation sequence concealed until participants were enrolled and assigned to interventions?		Y/PY/PN/N/NI	**Y**
1.3 Did baseline differences between intervention groups suggest a problem with the randomization process?	There were unequal groups (Treated *n* = 21 vs. Untreated *n* = 10); in addition, the baselines between *Treated* and *Untreated* eyes were unequal; “some concerns”.	Y/PY/PN/N/NI	**Y**
Risk-of-bias judgement	There is a risk-of-bias due to the small sample and this concern was identified the issue from the authors themselves (see Russell, 2017, page 858).	Low/High/Some concerns	**Low/Some concerns**
Optional: What is the predicted direction of bias arising from the randomization process?	Favours expirmental; change of LogMAR, ambulatory navigation/mobility and full-field sensitivity improves visual function	NA/Favours experimental/Favours comparator/Towards null/Away from null/Unpredictable	**Favours expirmental**
**Domain 2: Risk of bias due to deviations from the intended interventions (*effect of assignment to intervention*)**			
**Signalling questions**	**Comments**	**Response options**	**Actual responses—Russell et al., 2017**
2.1. Were participants aware of their assigned intervention during the trial?	The study was “open-label” therefore the patients were unblinded.	Y/PY/PN/N/NI	**N**
2.2. Were carers and people delivering the interventions aware of participants’ assigned intervention during the trial?		Y/PY/PN/N/NI	**N**
2.3. If Y/PY/NI to 2.1 or 2.2: Were there deviations from the intended intervention that arose because of the trial context?	Deviations may arise on the basis of being unblinded due to the “trial context”, specifcally the absence of unequal baselines) (see interpretation in the Cochrane advice, Rob-2 explanation no. 2.3)	NA/Y/PY/PN/N/NI	**PY**
2.4 If Y/PY to 2.3: Were these deviations likely to have affected the outcome?	There is “NI”; there was no trial protocol avabilable in the Russell paper, or no IND 13804 document or not included in the BLA 125610 (FDA)	NA/Y/PY/PN/N/NI	**NI**
2.5. If Y/PY/NI to 2.4: Were these deviations from intended intervention balanced between groups?	See question above, 2.4	NA/Y/PY/PN/N/NI	**NI**
2.6 Was an appropriate analysis used to estimate the effect of assignment to intervention?		Y/PY/PN/N/NI	**Y**
2.7 If N/PN/NI to 2.6: Was there potential for a substantial impact (on the result) of the failure to analyse participants in the group to which they were randomized?		NA/Y/PY/PN/N/NI	**N**
Risk-of-bias judgement		Low/High/Some concerns	**Some concerns**
Optional: What is the predicted direction of bias due to deviations from intended interventions?		NA/Favours experimental/Favours comparator/Towards null/Away from null/Unpredictable	**Favours expirmental**
**Domain 3: Missing outcome data**			
**Signalling questions**	**Comments**	**Response options**	**Actual responses—Russell et al., 2017**
3.1 Were data for this outcome available for all, or nearly all, participants randomized?		Y/PY/PN/N/NI	**Y**
3.2 If N/PN/NI to 3.1: Is there evidence that the result was not biased by missing outcome data?		NA/Y/PY/PN/N	**N**
3.3 If N/PN to 3.2: Could missingness in the outcome depend on its true value?		NA/Y/PY/PN/N/NI	**N**
3.4 If Y/PY/NI to 3.3: Is it likely that missingness in the outcome depended on its true value?		NA/Y/PY/PN/N/NI	**N**
Risk-of-bias judgement		Low/High/Some concerns	**Low**
Optional: What is the predicted direction of bias due to missing outcome data?		NA/Favours experimental/Favours comparator/Towards null/Away from null/Unpredictable	**Favours expirmental**
**Domain 4: Risk of bias in measurement of the outcome**			
**Signalling questions**	**Comments**	**Response options**	**Actual responses—Russell et al., 2017**
4.1 Was the method of measuring the outcome inappropriate?	The outcome methods for the mobility test (MLMT) had several measurements rolled into one final outcome without sufficient data incuding: (i) speed; (ii) time; (iii) accuracy; (iv) obstacles; (v) time penalties; (vi) lux, and; (vii) the scales of ordinal vs. logarithmic interpretation. Consequently, (a) the full direct data and measurements were not avaiable, and; (b) the final outcome may have different interpretations; see comments from FDA reviewers in the BLA 125610.	Y/PY/PN/N/NI	**Y**
4.2 Could measurement or ascertainment of the outcome have differed between intervention groups?	As above.	Y/PY/PN/N/NI	**PY**
4.3 If N/PN/NI to 4.1 and 4.2: Were outcome assessors aware of the intervention received by study participants?	Open-label, therefore there was no blinding.	NA/Y/PY/PN/N/NI	**Y**
4.4 If Y/PY/NI to 4.3: Could assessment of the outcome have been influenced by knowledge of intervention received?	The study is an open-label, unblinded and approved design; while there may be some unconscious influence, there is no evdience for such, therefore was no influence reported; PN or N.	NA/Y/PY/PN/N/NI	**PN/N**
4.5 If Y/PY/NI to 4.4: Is it likely that assessment of the outcome was influenced by knowledge of intervention received?		NA/Y/PY/PN/N/NI	**PN/N**
Risk-of-bias judgement		Low/High/Some concerns	**Some concerns**
Optional: What is the predicted direction of bias in measurement of the outcome?		NA/Favours experimental/Favours comparator/Towards null/Away from null/Unpredictable	**Favours expirmental**
**Domain 5: Risk of bias in selection of the reported result**			
**Signalling questions**	**Comments**	**Response options**	**Actual responses—Russell et al., 2017**
5.1 Were the data that produced this result analysed in accordance with a pre-specified analysis plan that was finalized before unblinded outcome data were available for analysis?	There was no pre-specified analysis plan (a protocol) so there is: NI.	Y/PY/PN/N/NI	**NI**
Is the numerical result being assessed likely to have been selected, on the basis of the results, from...	The MLMT assay is a novel primary outcome; see question and response above in 4.2	Y/PY/PN/N/NI	**NI**
5.2 … multiple eligible outcome measurements (e.g., scales, definitions, time points) within the outcome domain?		Y/PY/PN/N/NI	**NI**
5.3 … multiple eligible analyses of the data?		Y/PY/PN/N/NI	
Risk-of-bias judgement		Low/High/Some concerns	**Some concerns**
Optional: What is the predicted direction of bias due to selection of the reported result?		NA/Favours experimental/Favours comparator/Towards null/Away from null/Unpredictable	**Favours experimental**
**Overall risk of bias**			
	**Comments**	**Response options**	**Actual responses—Russell et al., 2017**
**Risk-of-bias judgement**	Further data would be valauble to conclude an overall risk of bias/judgement.	Low/High/Some concerns	**Some concerns**
Optional: What is the overall predicted direction of bias for this outcome?		NA/Favours experimental/Favours comparator/Towards null/Away from null/Unpredictable	**Favours experimental**

### Appendix A.7. PRISMA—Structured Summary

**Title** A systematic review and meta-analyses of interventional clinical trial studies for gene therapies for the Inherited Retinal Degenerations (IRDs)

**Abstract**

**Background**

Retinitis pigmentosa (RP) is a group of inherited retinal degenerations (IRDs) causing the deterioration of rod and cone photoreceptor cells in the retina leading to visual impairment or blindness. There is currently no cure for RP. Leber Congenital Amaurosis (LCA), a juvenile form of RP, shows an early infant-onset form of the disease characterised by severe retinal dystrophy, vision loss, nystagmus and an almost non-recordable ERG. Gene therapy is a potential treatment for LCA with previously published trials aimed at addressing several clinical outcomes, including visual acuity, mobility, visual field testing and retinal thickness, amongst others.

**Objectives**

To conduct a systematic review of interventional clinical trial studies for RP and to assess and compare the effectiveness of available gene therapy treatments.

**Search methods**

Ovid databases were searched in MEDLINE (from Jan. 1946 to Jun. 2020), EMBASE (Jan. 1980 to Jun. 2020), FDA and ClinicalTrials.gov.

**Selection criteria**

Randomised controlled trials (RCTs) and non-randomised studies of the effects of interventions (NRSIs) with any gene therapy treatment for any human patients diagnosed with any syndromic or non-syndromic RP.

**Data collection and analysis**

Standard methodological procedures of the Cochrane Collaboration for screening, data abstraction, and study assessment. A structured PICOS search strategy used screened records and abstracted data following with a review and assessment of risk-of-bias tools with included studies (with two independent authors). 115 records were found, 7 articles were duplicated and removed, 108 publications were screened, 87 were excluded and 21 articles were accessed for eligibility; of these, 15 articles were excluded: one (1) was not applicable (choroideremia), five (5) were follow-up studies and nine (9) articles included duplicate data. A final six (6) primary articles were conducted for review and meta-analyses, summarised in a PRISMA flowchart.

**Main results**

Six (6) clinical trial studies reported one (1) RCT and five (5) NRSIs, including a sample of *n* = 84 LCA2 (RPE65) patients across three countries (UK, France and the USA). A gene therapy augmentation treatment for recombinant AAV-RPE65 was sub-retinally transfected for a range of subjects spanning ages from 4–44 years. Twenty-three (23) outcomes were assessed and only five (5) outcomes were reported for meta-analyses: visual acuity (logMAR), ambulatory navigation/mobility, full-field stimulus (FST) testing (red wavelength) measuring log10(cd.s/m^2^), full-field stimulus (FST) testing (blue wavelength) measuring log10(cd.s/m^2^), and a final measurement of retinal thickness (evaluating OCT at the fovea). Twelve meta-analyses were reported but only one (1) assay, visual acuity (VA), was common to all six (6) papers.

ETDRS logMAR results found a summary weighted mean difference (MD) of −0.06 logMAR improvement over treated vs. untreated eyes (95% CI −0.14, 0.02), *p* = 0.16, including six (6) studies with a I^2^ heterogeneity of 65%. An ambulatory navigation/mobility test across a light-intensity level of 4 lux showed an RR (risk ratio) improvement of 1.03, over treated vs. untreated eyes (95% CI 0.75, 1.42), *p* = 0.84, including four studies with a I^2^ heterogeneity of 0%. A summary weighted mean difference (MD) of FST (full-field stimulus testing) (red) showed 0.89 log10(cd.s/m^2^) over treated vs. untreated eyes (95% CI −0.06, 1.84), *p* = 0.07, and; a summary weighted mean difference (MD) of FST (blue) showed 1.69 log10(cd.s/m^2^) over treated vs. untreated eyes (95% CI 1.21, 2.16), *p* = 0.00001. Finally, an RR improvement of retinal thickness was 1.15 (95% CI 0.45, 3.00), *p* = 0.77. Of the 12 meta-analyses, only three of the meta-analyses met statistical significance: FST (red light) with an RR improvement of 1.89 (95% CI 1.04, 3.41), *p* = 0.04; FST (blue light) with a MD improvement of 1.69 highlighted above, with a *p* = 0.00001, and finally; FST (blue light) reported an RR improvement of 2.01, (95% CI 1.32, 3.06), *p* = 0.001. All other assays did not reach statistical significance.

Study design quality and an overall risk-of-bias judgement showed “*Low/Moderate*” and “*Towards to null/Unpredictable*” with the ROBIN-I tool (NRSIs), and “*Some concerns*” and “*Favours experimental*” with the RoB-2 tool (RCT).

**Conclusions**

The objective of this work was to conduct a systematic review of interventional clinical trial studies for IRDs and to assess and compare the effectiveness of available gene therapy treatments. Following the search, review and analysis of the relevant studies, the systematic review concluded that a meta-analysis for AAV-RPE65 gene therapy for LCA2 reported a modest improvement for visual acuity, mobility and full-field stimulus testing (FST). However, other than FST, there was no clinically meaningful benefit and no statistical significance from the six collected studies. One RCT found a clinically meaningful benefit for an assessment for a primary endpoint for mobility, a MD of 1.6 (95% CI 0.72–2.41), *p* = 0.0013.

In terms of a recommendation to support the IRD patient communities and researchers, we propose that full and open-access data is key. If the field is to be progressed and improved, then objective and transparent results need to be shared in order to improve outcomes, analysis, reporting and interpretation.

### Appendix A.8. PRISMA List for LCA2 Studies

**Table A4:** PRISMA List for LCA2 Studies.

Section/Topic	#	Checklist Item	Reported on Page #
**TITLE**			
Title	**1**	Identify the report as a systematic review, meta-analysis, or both.	**Page 1**
**ABSTRACT**			
Structured summary	**2**	Provide a structured summary including, as applicable: background; objectives; data sources; study eligibility criteria, participants, and interventions; study appraisal and synthesis methods; results; limitations; conclusions and implication ns of key findings; systematic review registration number.	**Page 53, (Appendix A.7)**
**BACKGROUND**			
Rationale	**3**	Describe the rationale for the review in the context of what is already known.	**Page 1–2 (Introduction)**
Objectives	**4**	Provide an explicit statement of questions being addressed with reference to participants, interventions, comparisons, outcomes, and study design (PICOS).	**Page 2 (Materials and Methods); page 4 (Results); Table S1 (Supplemental).**
**METHODS**			
Protocol and registration	**5**	Indicate if a review protocol exists, if and where it can be accessed (e.g., Web address), and, if available, provide registration information including registration number.	**Informal proposal/protocol assessed and peer-reviewed at the University of Edinburgh.**
Eligibility criteria	**6**	Specify study characteristics (e.g., PICOS, length of follow-up) and report characteristics (e.g., years considered, language, publication status) used as criteria for eligibility, giving rationale.	**Table S1 (Supplemental); Appendix A.4.**
Information sources	**7**	Describe all information sources (e.g., databases with dates of coverage, contact with study authors to identify additional studies) in the search and date last searched.	**Table S1 (Supplemental); Appendix A.1. page 15.**
Search	**8**	Present full electronic search strategy for at least one database, including any limits used, such that it could be repeated.	**Appendix A.1. page 15; Appendix A.2, page 21.**
Study selection	**9**	State the process for selecting studies (i.e., screening, eligibility, included in systematic review, and, if applicable, included in the meta-analysis).	**Appendix A.2, page 21; Appendix A.3, page 31.**
Data collection process	**10**	Describe method of data extraction from reports (e.g., piloted forms, independently, in duplicate) and any processes for obtaining and confirming data from investigators.	**Page 4 (Materials and Methods).**
Data items	**11**	List and define all variables for which data were sought (e.g., PICOS, funding sources) and any assumptions and simplifications made.	**2 NRSI studies were supported by academic funding (Jacobson 2012, Le Meur 2018); 1 NRSI was supported by both academic and industry funding (Bainbridge 2015); 2 NRSI studies were supported by industry funding (Testa 2013, Weleber 2016); 1 RCT was supported by industry funding (Russell 2017).**
Risk of bias in individual studies	**12**	Describe methods used for assessing risk of bias of individual studies (including specification of whether this was done at the study or outcome level), and how this information is to be used in any data synthesis.	**Page 4 (Materials and Methods); Appendix A.5 and Appendix A.6.**
Summary measures	**13**	State the principal summary measures (e.g., risk ratio, difference in means).	**Page 4 (Materials and Methods).**
Synthesis of results	**14**	Describe the methods of handling data and combining results of studies, if done, including measures of consistency (e.g., I2) for each meta-analysis.	**Page 4 (Materials and Methods); Page 8–16.**
Risk of bias across studies	**15**	Specify any assessment of risk of bias that may affect the cumulative evidence (e.g., publication bias, selective reporting within studies).	**Page 4 (Materials and Methods); Appendix A.5 and Appendix A.6.**
Additional analyses	**16**	Describe methods of additional analyses (e.g., sensitivity or subgroup analyses, meta-regression), if done, indicating which were pre-specified.	**n/a**
**RESULTS**			
Study selection	**17**	Give numbers of studies screened, assessed for eligibility, and included in the review, with reasons for exclusions at each stage, ideally with a flow diagram.	**Page 5, Figure 1 (Results)**
Study characteristics	**18**	For each study, present characteristics for which data were extracted (e.g., study size, PICOS, follow-up period) and provide the citations.	**Table S1 (Supplemental); Appendix A.2.**
Risk of bias within studies	**19**	Present data on risk of bias of each study and, if available, any outcome level assessment (see item 12).	**Appendix A.5 and Appendix A.6.**
Results of individual studies	**20**	For all outcomes considered (benefits or harms), present, for each study: (a) simple summary data for each intervention group (b) effect estimates and confidence intervals, ideally with a forest plot.	**Table 3, page 13.**
Synthesis of results	**21**	Present results of each meta-analysis done, including confidence intervals and measures of consistency.	**Table 3, page 12.**
Risk of bias across studies	**22**	Present results of any assessment of risk of bias across studies (see Item 15).	**Table 2, page 11**
**DISCUSSION**			
Summary of evidence	**24**	Summarize the main findings including the strength of evidence for each main outcome; consider their relevance to key groups (e.g., healthcare providers, users, and policy makers).	**Page 15 (Conclusion); Appendix A.7 (Structured summary).**
Limitations	**25**	Discuss limitations at study and outcome level (e.g., risk of bias), and at review-level (e.g., incomplete retrieval of identified research, reporting bias).	**Table 2, Page 11; Appendix A.5 and Appendix A.6.**
Conclusions	**26**	Provide a general interpretation of the results in the context of other evidence, and implications for future research.	**Page 15 (Conclusion); Table S4a,b.**
**FUNDING**			
Funding	**27**	Describe sources of funding for the systematic review and other support (e.g., supply of data); role of funders for the systematic review.	**None.**
