# Supplementary material for: A Systematic Review and Meta-Analyses of Interventional Clinical Trial Studies for Gene Therapies for the Inherited Retinal Degenerations (IRDs)
Source: Biomolecules. 2021 May 19;11(5):760. doi: 10.3390/biom11050760 (PMC8160708; doi:10.3390/biom11050760)
Supplement: Supplementary file 1 [file biomolecules-11-00760-s001.zip › biomolecules-1157391-supplementary.pdf]

Supplementary Figures and Tables.

Figures:

- **Figure S.1. (Supplemental).** (a) A simple hierarchy of evidence; (b) A list of 23 assays from the 6 selected studies; (c) Each of the 23 assays re-grouped and colour coded to clearly distinguish how the specific assays were to be used in the study
- **Figure S.2 (Supplemental).** Visual acuity logMAR, with a random effects model and summary statistic for dichotomous data showed

Tables

- **Table S.1 (Supplemental).** PICOS results. The PICOS search terms, keywords, MeSH terms, search strings and Boolean operators were used and identified in Materials & Method (using Ovid Database), additionally defined Appendix A.1 to Appendix A.3.
- **Table S.2 (Supplemental).** All mean difference (MD) values for all visual acuity logMAR changes across all six (6) papers. All data was retrieved and analysed by two independent authors.
- **Table S.3 (Supplemental).** All ambulatory navigation / mobility across all six (6) papers. All data was retrieved and analysed by two independent authors.
- **Table S.4 (Supplemental).** MLMT (Russell et al. 2017) and FDA (BLA No. 125610).

Figure S.1 (Supplemental)

(a) A simple hierarchy of evidence for assessing outcome assays in LCA2 interventional studies, modelled in the hierarchy of evidence for assessing the quality of evidence in clinical research studies (Lang ES, 2007)[1]

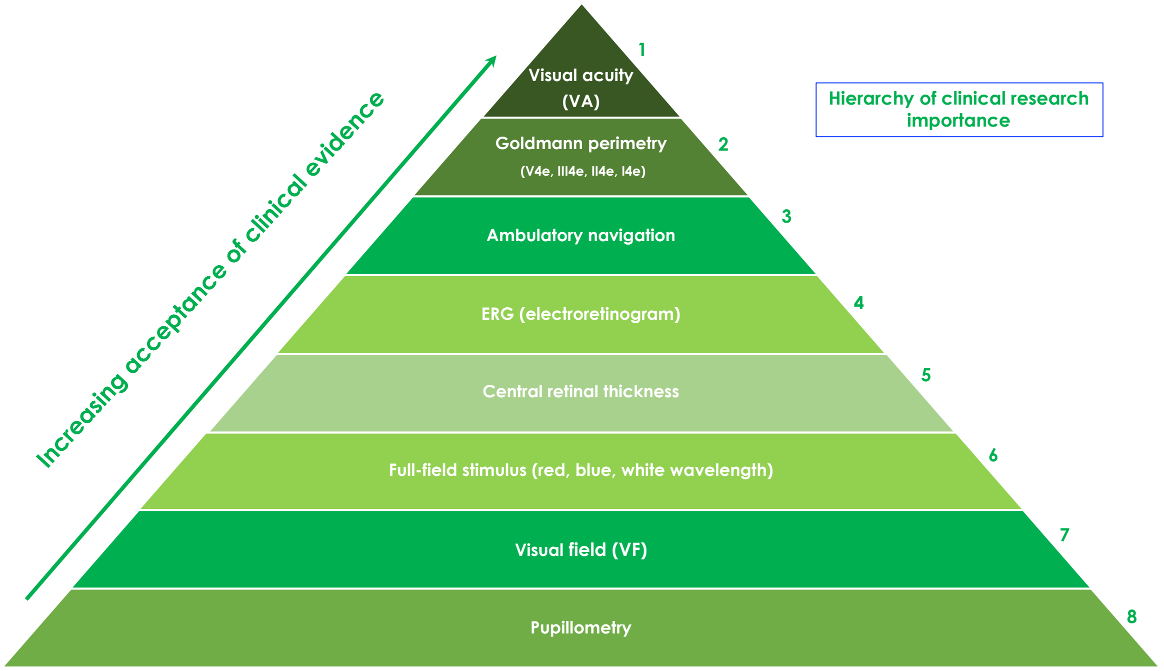

(b) Each of the 23 assays were ranked in the order of most prevalent assays chosen from the 6 selected studies.

No.	Assays	Most prevalent assays across the six studies
1	Visual acuity (VA)	6
2	Goldmann perimetry (V4e, III4e, II4e, I4e)	5
3	Ambulatory navigation	4
4	ERG (electroretinogram)	3
5	Retinal thickness, OCT (CRT / fovea)	3
6	FAF (fundus autofluorescence)	2
7	FST (blue wavelength) log10(cd.s/m^2	2
8	FST (red wavelength) log10(cd.s/m^2	2
9	Nystagmus testing	2
10	Pupillary light reflex (PLR)	2
11	Pupillometry	2
12	Dark-adapted perimetry	2
13	Colour vision	1
14	Contrast sensitivity	1
15	fMRI	1
16	FST (white wavelength) log10(cd.s/m^2	1
17	Fundus near infrared	1
18	Humphrey VF, fovea sensitivity	1
19	Humphrey VF, macula threshold	1
20	Microperimetry	1
21	Spectral sensitivity	1
22	Vision questionnaire (QoL)	1
23	Visual field (VF) (V_{30} / V_{TOT})	1

(c) Each of the 23 assays were re-grouped and colour coded to clearly distinguish how the specific assays and were to be used in the study. For example, in Group 1, five (1 to 5) assays were capable to be used as a meta-analysis; while, in Group 2, seven (6 to 12) assays were assessed individually, but were not capable for generating a meta-analysis.

		Outcome assays for meta-analysis	Most prevalent assays	Study authors
Group 1	1	Visual acuity (BCVA) (logMAR)	6	Bainbridge, Jacobson, Le Meur, Russell, Testa, Weleber
	2	Ambulatory navigation / mobility	4	Bainbridge, Jacobson, Russell, Testa
	3	Retinal thickness, OCT (CRT / fovea)	3	Bainbridge, Jacobson
	4	FST (blue) log10(cd.s/m^2)	2	Jacobson, Russell
	5	FST (red) log10(cd.s/m^2)	2	Jacobson, Russell
Group 2	6	Goldmann perimetry (V4e, III4e, II4e, I4e)	5	Bainbridge, Le Meur, Russell, Testa, Weleber
	7	ERG (electroretinogram)	3	Bainbridge, Testa, Weleber
	8	FAF (fundus autofluorescence)	2	Bainbridge, Testa
	9	Nystagmus testing	2	Le Meur, Testa
	10	Pupillary light reflex (PLR)	2	Le Meur, Testa
	11	Pupillometry	2	Jacobson, Le Meur
	12	Dark-adapted perimetry	2	Bainbridge, Testa
Group 3	13	Colour vision	1	Bainbridge
	14	Contrast sensitivity	1	Bainbridge
	15	fMRI	1	Le Meur
	16	FST (white) log10(cd.s/m^2)	1	Russell
	17	Fundus near infrared	1	Jacobson
	18	Humphrey VF, fovea sensitivity	1	Russell
	19	Humphrey VF, macula threshold	1	Russell
	20	Microperimetry	1	Le Meur
	21	Spectral sensitivity	1	Bainbridge
	22	Vision questionnaire (QoL)	1	Weleber
	23	Visual field (VF) (V_{30}/ V_{TOT})	1	Weleber

Figure S.2 (Supplemental) Visual acuity (dichotomous analysis)

Study or Subgroup	Treated Eye Events	Treated Eye Total	Untreated Eye Events	Untreated Eye Total	Weight	Risk Ratio M-H, Random, 95% CI
Bainbridge 2015	7	12	7	12	20.1%	1.00 [0.51, 1.97]
Le Meur 2018	3	9	3	9	5.4%	1.00 [0.27, 3.69]
Russell 2017	18	20	5	9	25.4%	1.62 [0.89, 2.96]
Testa 2013	5	5	4	5	33.9%	1.22 [0.73, 2.06]
Weleber 2016	5	12	8	12	15.1%	0.63 [0.29, 1.36]
**Total (95% CI)**		**58**		**47**	**100.0%**	**1.13 [0.83, 1.53]**
Total events	38		27			

Heterogeneity: $Tau^2 = 0.00$; $Chi^2 = 3.92$, df = 4 (P = 0.42); $I^2 = 0\%$
Test for overall effect: Z = 0.77 (P = 0.44)

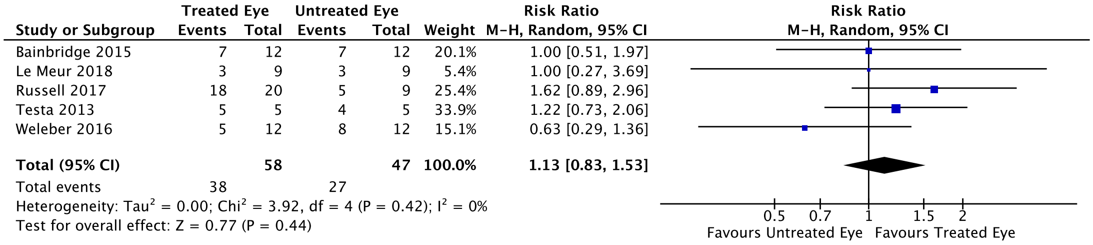

Figure S.2 (Supplemental). Visual acuity logMAR, with a random effects model and summary statistic for dichotomous data showed that the line of no effect (RR of 1.13 (95% CI 0.83, 1.53)) indicated an improvement with treated eyes compared with untreated eyes and this supports the previous continuous data in the marginal difference of -0.06 logMAR (main paper Figure 2). The dichotomous data indicated a small improvement that did not reached clinical significance.

Table S.1(Supplemental). PICOS results. The PICOS search terms, keywords, MeSH terms, search strings and Boolean operators were used and identified in Materials & Method (using Ovid Database), additionally defined within Appendix A.1 to Appendix A.3.

PICOS	Lead search terms *(Ovid Database)*	Number of search terms	MEDLINE Database Results	EMBASE Database Results	Cochrane Database Results	Additional resources
**P**opulation	Retinitis pigmentosa, and other terms	8	85,822	867,550	7	-
**I**ntervention	Gene therapies, and other terms	8	80,450	138,881	0	-
**C**omparison	Comparison (no terms)	0	0	0	6	-
**O**utcomes	Visual acuity, and other terms	15	267,300	520,911	8	2
**S**tudy design	Clinical trial, and other terms	5	702,125	792,290	8	-
	Boolean results	**36**	**58**	**55**	**0**	**2**
	**Total results**		**115**			

Table S.2 (Supplemental). All mean difference (MD) values for all visual acuity logMAR changes across all six (6) papers. All data was retrieved and analysed by two independent authors.

LCA2-RPE65 gene therapy for mean differences of visual acuty (VA) outcomes (logMAR)

Author / Year	*Treated eye*		*Control eye*		Mean Difference (logMAR)	VA Improves / Deteriorates
	Treated eye (n=12)		*Control eye (n=12)*			
**Bainbridge 2015 (1 year)**	**Mean**	**SD**	**Mean**	**SD**		
Baseline	0.776	0.316	0.703	0.335		
1 Year	0.768	0.314	0.640	0.361		
**Change of logMAR**	**-0.008**	[0.193]	**-0.063**	[0.121]	**0.055**	VA deteriorates
	Treated eye (n=12)		*Control eye (n=12)*			
**Bainbridge 2015 (3 year)**	**Mean**	**SD**	**Mean**	**SD**		
Baseline	0.776	0.316	0.703	0.335		
3 Year	0.848	0.386	0.690	0.395		
**Change of logMAR**	**0.072**	0.263	**-0.013**	0.139	**0.084**	VA deteriorates
	Treated eye (n=15)		*Control eye (n=15)*			
**Jacobson 2012 (3 year)**	**Mean**	**SD**	**Mean**	**SD**		
Baseline	1.090	0.426	0.960	0.503		
3 Year	0.970	0.426	0.910	0.503		
**Change of logMAR**	**-0.120**	0.194	**-0.050**	0.077	**-0.070**	VA improves
	Treated eye (n=9)		*Control eye (n=9)*			
**Le Meur 2018 (1 year)**	**Mean**	**SD**	**Mean**	**SD**		
Baseline	1.070	0.458	0.878	0.413		
1 Year	1.020	0.389	0.858	0.358		
**Change of logMAR**	**-0.050**	0.187	**-0.020**	0.122	**-0.030**	VA improves
	Treated eye (n=9)		*Control eye (n=9)*			
**Le Meur 2018 (2-3 year)**	**Mean**	**SD**	**Mean**	**SD**		
Baseline	1.070	0.458	0.878	0.413		
2-3 Year	1.010	0.451	0.910	0.480		
**Change of logMAR**	**-0.057**	0.152	**0.0325**	0.141	**-0.089**	VA improves
	Treated (n=20)		*Control (n=9)*			
**Russell 2017**	**Mean**	**SD**	**Mean**	**SD**		
Baseline	1.137	0.369	0.987	0.306		
1 Year	0.975	0.542	0.954	0.333		
**Change of logMAR**	**-0.163**	0.336	**-0.031**	0.097	**-0.131**	VA improves
	Treated eye (n=5)		*Control eye (n=5)*			
**Testa 2013 (1 year)**	**Mean**	**SD**	**Mean**	**SD**		
Baseline	1.470	0.316	1.136	0.335		
1 Year	0.984	0.502	0.872	0.464		
**Change of logMAR**	**-0.486**	0.040	**-0.264**	0.130	**-0.222**	VA improves
	Treated eye (n=5)		*Control eye (n=5)*			
**Testa 2013 (3 year)**	**Mean**	**SD**	**Mean**	**SD**		
Baseline	1.470	0.316	1.136	0.335		
3 Year	1.0200	0.471	0.9680	0.551		
**Change of logMAR**	**-0.450**	0.094	**-0.168**	0.238	**-0.282**	VA improves
	Treated eye (n=12)		*Control eye (n=12)*			
**Weleber 2016 (1 year)**	**Mean**	**SD**	**Mean**	**SD**		
Baseline	1.220	0.474	1.131	0.514		
1 Year	1.195	0.558	1.085	0.541		
**Change of logMAR**	**-0.025**	0.133	**-0.046**	0.073	**0.021**	VA deteriorates

Table S.3 (Supplemental). All ambulatory navigation / mobility across all six (6) papers. All data was retrieved and analysed by two independent authors. Ratios may differ from RevMan analysis, partially computed by weightings.

Sub-group A: Ambulatory navigation / mobility, lux 4 only (common to 4 studies)

	Treated eyes			Untreated eyes			
	Events	Total	Percentage of positive events	Events	Total	Percentage of positive events	Ratio
Bainbridge 2015	2	11		0	11		5.00
Jacobson 2012	4	6		3	6		1.33
Russell 2017	3	20		2	9		0.68
Testa 2013	5	5		5	5		1.00
Sum of Events, Total	**14**	**42**	**33.3%**	**10**	**31**	**32.3%**	**2.00**

Sub-group B: Ambulatory navigation / mobility, low ambient light (lux 0.2, 0.6, 1, 2, 4)

	Treated eyes			Untreated eyes			
	Events	Total	Percentage of positive events	Events	Total	Percentage of positive events	Ratio
Bainbridge 2015	2	11		0	11		5.00
Jacobson 2012	20	24		17	24		1.18
Russell 2017	16	20		2	9		3.60
Testa 2013	5	5		5	5		1.00
Sum of Events, Total	**43**	**60**	**71.7%**	**24**	**49**	**49.0%**	**2.69**

Sub-group C: Ambulatory navigation / mobility, high ambient light (lux 10, 15, 50, 100)

	Treated eyes			Untreated eyes			
	Events	Total	Percentage of positive events	Events	Total	Percentage of positive events	Ratio
Bainbridge 2015	0	12		2	12		0.20
Jacobson 2012	0	6		2	6		0.20
Russell 2017	3	20		2	9		0.68
Sum of Events, Total	**3**	**38**	**7.9%**	**6**	**27**	**22.2%**	**0.36**

Sub-group D: Ambulatory navigation / mobility, all ambient light (lux 0.2, 0.6, 1, 2, 4, 10, 15, 50, 100)

	Treated eyes			Untreated eyes			
	Events	Total	Percentage of positive events	Events	Total	Percentage of positive events	Ratio
Bainbridge 2015	2	11		2	12		1.09
Jacobson 2012	20	30		19	30		1.05
Russell 2017	19	20		4	9		2.14
Testa 2013	5	5		5	5		1.00
Sum of Events, Total	**46**	**66**	**69.7%**	**30**	**56**	**53.6%**	**1.32**

Table S.4 (Supplemental). MLMT (Russell et al. 2017) and FDA (BLA No. 125610).

Table S.4 (a). Data has been derived from the MLMT assay from Russell et al., (2017), Supplementary Appendix[2]. This study presents a "*Baseline Passing Level (lux level)*" to compare against a "*1 Year Passing Level (lux level)*", providing a "*Change score*". The change score requires a separate table (Chung et al., (2018) [6]) to identify data to convert a logarithmic scale to an ordinal scale and then compute a mean difference [MD] of 1.6, 95% CI 0.72-2.41, p = 0.0013.

Derived from Russell 2017, Supplementary appendix, pg. 11-13, Table S3. Individual Subject-Level Data (Primary Endpoint, MLMT)								
Number	Subject ID	Age at randomization	Gender	Baseline Passing Level (lux level)	Baseline MLMT score	! Year Passing Level (lux level)	1 yr timepoint MLMT score	Change Score
**Treatment (n=20)**								
1	CH-22	20	F	50	3	1	6	3
2	IA-23	11	F	10	4	1	6	2
3	CH-25	33	F	>400	-1	>400	-1	0
4	IA-26	4	F	125	2	1	6	4
5	IA-27	11	F	50	3	1	6	3
6	CH-30	6	M	4	5	1	6	1
7	CH-31	13	F	50	3	1	6	3
8	IA-32	5	F	10	4	1	6	2
9	IA-33	6	F	4	5	1	6	1
10	CH-34	13	M	10	4	1	6	2
11	IA-35	34	M	50	3	10	4	1
12	IA-38	16	M	50	3	4	5	2
13	CH-41	6	F	50	3	1	6	3
14	CH-42	6	F	10	4	4	5	1
15	CH-44	5	M	10	4	4	5	1
16	CH-45	11	M	50	3	1	6	3
17	CH-47	8	M	4	5	1	6	1
18	IA-49	34	F	125	2	50	3	1
19	IA-50	44	F	125	2	10	4	2
20	IA-52	5	M	4	5	1	6	1
					3.3		5.2	1.85
**Control n=9**								
1	CH-16	10	F	50	3	10	4	1
2	CH-17	19	F	125	2	250	1	-1
3	CH-18	9	F	10	4	4	5	1
4	CH-19	7	M	50	3	125	2	-1
5	IA-29	28	F	50	3	50	3	0
6	CH-36	9	F	10	4	50	3	-1
7	CH-37	24	M	4	5	4	5	0
8	CH-43	4	M	50	3	4	5	2
9	CH-53	18	F	50	3	10	4	1
					3.3		3.6	0.22

Key - Scoring code for multi-lumiance mobility test change score (Chung et al 2018).

Lux	1	4	10	50	100 / 150	200 / 250	400	>400
Score	6	5	4	3	2	1	0	-1

The MLTM assay methodology was set out in Russell *et al*.[2], more comprehensively described by Chung *et al*.[6]. The MLMT score was calculated by taking into account several components – light intensity level (lux), accuracy, speed, time, obstacles, re-directions, collisions, faults, time penalties and a "final passing" score – however, these individual

components were not fully described in the Russell *et al.* paper [2], whereby the specific measurements were not available within the study, and not available within the FDA Biologics License Application. The Chung *et al.*[6], used the MLMT assay to describe a pool of 29 normal sighted subjects and 31 visually impaired IRD patient phenotypes, including twenty (20) subjects diagnosed with LCA, five (5) with choroideremia, four (4) with RP and one (1) each with Stargardt disease and Usher syndrome. Alternative models for mobility in other retinal degenerative disorders may be available in the literature [5, 7–11].

Independent criticism of the scoring system was ordinal (ranging from -1 to 6), while the light intensities (lux) that determined the scoring system was used in logarithmic scale, such that a two-point change in the ordinal scale may have a different interpretation depending on the baseline score (Darrow, 2019[4], (*Drug Discovery Today, Volume 4, Number 4, April 2019*).

Table S.4 (b). Further data has been derived from the MLMT results reported from the FDA (Zhu, Y-Y., 2017[12], BLA Clinical Review Memorandum No. 125610, *Figure 10 – MLMT Score Change for Individual Subjects Using Both Eyes (ITT), pp 51)*). This study presents that some of these patients have successfully navigated the MLMT result however, in the context of a small sample size population, 11 treated patients met the endpoint, and 9 treated patients missed the endpoint (55% vs 45%), in a single duration period (1-year timepoint). Any follow-up study for the same patients under the 2-year or 3-year timepoint, or for any phase IV data, may provide an independent valuable insight for the LCA field.

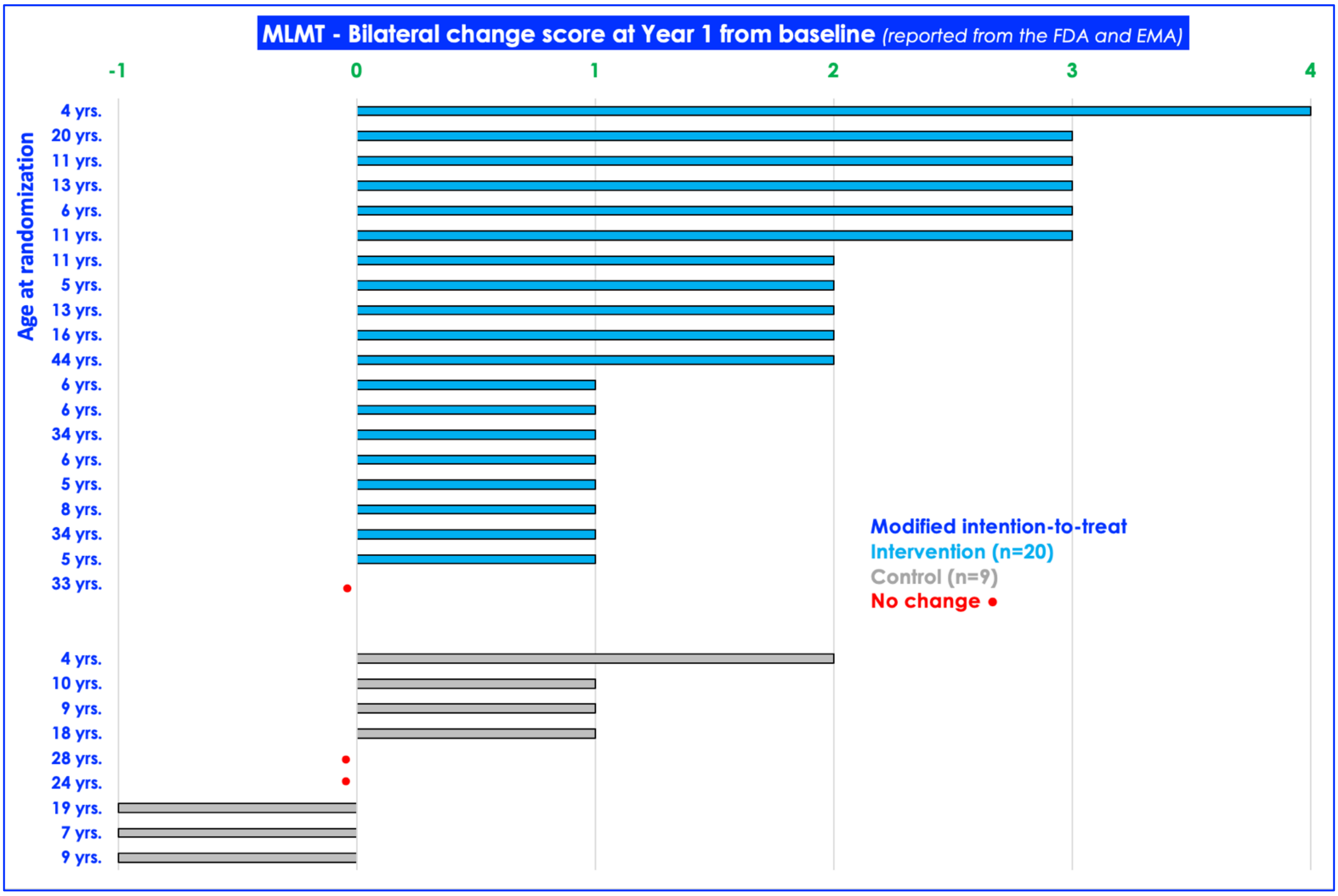

In summary, the overall result of the MLMT assay showed a benefit of 11 of the 20 treated patients (55%) met a clinically meaningful outcome, compared to 1 of 9 untreated patients (11%).

References

1. Lang, E.S.; Wyer, P.C.; Haynes, R.B. Knowledge Translation: Closing the Evidence-to-Practice Gap. *Ann. Emerg. Med.* **2007**, *49*, 355–363, doi:10.1016/j.annemergmed.2006.08.022.
2. Russell, S.; Bennett, J.; Wellman, J.A.; Chung, D.C.; Yu, Z.-F.; Tillman, A.; Wittes, J.; Pappas, J.; Elci, O.; McCague, S.; et al. Efficacy and safety of voretigene neparvovec (AAV2-hRPE65v2) in patients with RPE65 -mediated inherited retinal dystrophy: A randomised, controlled, open-label, phase 3 trial. *Lancet* **2017**, *390*, 849–860, doi:10.1016/s0140-6736(17)31868-8.
3. Weleber, R.G.; Pennesi, M.E.; Wilson, D.J.; Kaushal, S.; Erker, L.R.; Jensen, L.; McBride, M.T.; Flotte, T.R.; Humphries, M.; Calcedo, R.; et al. Results at 2 Years after Gene Therapy for RPE65-Deficient Leber Congenital Amaurosis and Severe Early-Childhood–Onset Retinal Dystrophy. *Ophthalmology* **2016**, *123*, 1606–1620, doi:10.1016/j.ophtha.2016.03.003.A
4. Darrow, J.J. Luxturna: FDA documents reveal the value of a costly gene therapy. *Drug Discov. Today* **2019**, *24*, 949–954, doi:10.1016/j.drudis.2019.01.019.

5. Ayton, L.N.; Rizzo, J.F.; Bailey, I.L.; Colenbrander, A.; Dagnelie, G.; Geruschat, D.R.; Hessburg, P.C.; McCarthy, C.D.; Petoe, M.A.; Rubin, G.S.; et al. Harmonization of Outcomes and Vision Endpoints in Vision Restoration Trials: Recommendations from the International HOVER Taskforce. *Transl. Vis. Sci. Technol.* **2020**, *9*, 25–89, doi:10.1167/tvst.9.8.25.
6. Chung DC, McCague S, Yu ZF, Thill S, DiStefano-Pappas J, Bennett J, Cross D, Marshall K, Wellman J, High KA. Novel mobility test to assess functional vision in patients with inherited retinal dystrophies. Clin Exp Ophthalmol. 2018 Apr;46(3):247-259. doi: 10.1111/ceo.13022. Epub 2017 Aug 31. PMID: 28697537; PMCID: PMC5764825.
7. Warrian, K.J.; Katz, L.J.; Myers, J.S.; Moster, M.R.; Pro, M.J.; Wizov, S.S.; Spaeth, G.L. A comparison of methods used to evaluate mobility performance in the visually impaired. *Br. J. Ophthalmol.* **2014**, *99*, 113–118, doi:10.1136/bjophthalmol-2014-305324.
8. Leat, S.J.; Lovie-Kitchin, J.E. Measuring mobility performance: Experience gained in designing a mobility course. *Clin. Exp. Optom.* **2006**, *89*, 215–228, doi:10.1111/j.1444-0938.2006.00050.x.
9. Haymes, S.; Guest, D.; Heyes, A.; Johnston, A. Comparison of Functional Mobility Performance with Clinical Vision Measures in Simulated Retinitis Pigmentosa. *Optom. Vis. Sci.* **1994**, *71*, 442–453, doi:10.1097/00006324-199407000-00004.
10. Geruschat, D.R.; Turano, K.A.; Stahl, J.W. Traditional Measures of Mobility Performance and Retinitis Pigmentosa. *Optom. Vis. Sci.* **1998**, *75*, 525–537, doi:10.1097/00006324-199807000-00022.
11. Hartong, D.T.; Jorritsma, F.F.; Neve, J.J.; Melis-Dankers, B.J.M.; Kooijman, A.C. Improved mobility and independence of night-blind people using night-vision goggles. *Investig. Ophthalmol. Vis. Sci.* **2004**, *45*, 1725–1731, doi:10.1167/iovs.03-1061.
12. Zhu, Y.-Y. BLA Clinical Review Memorandum-No. 125610 (FDA). 2017. Available online: https://www.fda.gov/files/vaccines%2Cblood%26biologics/published/Clinical-Review--December-16--2017---LUXTURNA.pdf (accessed on 8 March 2021).
